# Effects of season and antler growth on carcass traits and meat quality in wild male red deer harvested by selective stalking

**DOI:** 10.1371/journal.pone.0352456

**Published:** 2026-07-08

**Authors:** Martina Pérez Serrano, José Manuel Lorenzo, Roberto Bermúdez, Laura Purriños, Yolanda Fierro, Andrés José García, Datao Wang, Nicolás Alegría-Aravena, Louis Chonco, Tomás Landete-Castillejos

**Affiliations:** 1 Department of Agronomy Production, Higher Technical School of Agronomy, Food and Biosystems Engineering, UPM, Madrid, Spain; 2 Galician Meat Technology Centre, Galician Technology Park, Ourense, Spain; 3 Food Technology Department, Faculty of Science, University of Vigo, Ourense, Spain; 4 La Morera Game Estate, Abenójar, Spain; 5 Research Institute for Game Resources (IREC-CSIC-JCCM), University of Castilla-La Mancha, Albacete, Spain; 6 Game and Livestock Resources Section, Regional Development Institute (IDR), Castilla-La Mancha University, Albacete, Spain; 7 Institute for Health Research of Castilla-La Mancha (IDISCAM), Albacete, Spain; 8 Institute of Special Animal and Plant Sciences, Chinese Academy of Agricultural Sciences, Changchun, China; 9 Spanish Association Against Cancer (AECC)-Scientific Foundation AECC, Madrid, Spain; Universidade Federal de Mato Grosso do Sul, BRAZIL

## Abstract

Seasonal variation in temperate ecosystems influences food availability and the energetic demands associated with thermoregulation and reproduction, shaping physiological traits such as meat composition in wild animals. However, the specific effects of seasonality on meat quality in wild red deer remain insufficiently understood. This study evaluated how seasonality —represented by hunting month (September, January, April, and June)— affects the quality of the *Longissimus thoracis et lumborum* muscle in 32 adult wild male red deer harvested under minimal stress conditions. We hypothesized that seasonal fluctuations in diet and physiological requirements, particularly those linked to antler growth, would generate measurable differences in carcass traits and meat composition. Deer hunted in September (late summer) showed the highest carcass weight and yield, whereas the lowest values occurred in January (mid-winter) and June (early summer; p < 0.001). Intramuscular fat content was also greatest in September, with significant differences compared to April (p < 0.05). Additional seasonal effects were detected for pH at 72 h post-mortem (pH₇₂), shear force, and fatty acid profile (p < 0.05), indicating that muscle metabolism and textural properties vary across the year. Mineral composition exhibited a distinct pattern, suggesting a stronger association with skeletal mobilization during antler growth than with dietary changes. Mineral composition patterns were consistent with seasonal skeletal mobilization during antler growth: Ca and Mg were higher in April; Fe and Zn were lower in April and June. Overall, these findings demonstrate that both season and antler-growth effort affected meat quality and composition in wild male red deer, providing new insights into nutritional ecology and game meat quality.

## 1. Introduction

In recent years there has been a growing interest in alternative protein sources such as meat derived from non-farmed animal species. Red deer (*Cervus elaphus*) are a major large game species hunted in Europe, as they are widely distributed and highly abundant. Thus, they constitute probably the main game meat worldwide, and consumption has increased over the last decade [1,2], particularly in touristic cities in central and northern Europe. Venison from wild or estate culls has a lower environmental impact and carbon fingerprint than meat from industrial livestock systems, helping to maintain biodiversity conservation and rural development [3].

Season affects cervid meat quality, particularly in seasonal breeders [4–10]. In deer, most trophy hunting coincides with their rutting season. In addition, season of the year is one of the most influential factors for wild animals, as it affects food availability through plant productivity cycles, as well as other aspects of their biology. Deer hunting period also influences growth [5,6], carcass traits [7], and meat quality [7–10].

The effect of seasonality in male deer has a unique feature because of the huge physiological effort to grow antlers. Antlers are the fastest-growing mammalian tissue and can represent up to 28% of skeletal mass. Antler mineralization induces a process known as cyclical physiological osteoporosis, where minerals are mobilized from the skeleton to support antler development (reviewed in Landete-Castillejos et al. [11]). This process may influence the mineral profile of meat, particularly in spring-hunted animals [10,12,13]. In addition to seasonal effects, the type of hunting is also likely to influence meat characteristics. The most common forms of hunting deer in Spain are the traditional type known as Monteria (in which deer are chased by dogs towards the hunter’s line, thus producing a stressful death), or non-stressful death by selective shooting to reduce population density or to get particularly good trophies. This last hunting method form is known as stalking and implies a sudden low stress death (which is also common in most countries). Our research group found differences in deer meat quality between the winter driven hunt and non-stressful summer stalking that may be attributed to the level of ante mortem stress in the case of variables such as pH [12]. However, the effects observed for fat and mineral composition of meat seems to be seasonal, depending respectively on diet or cyclic osteoporosis in males. Therefore, the aim of this work was to study the effects of hunting month of adult wild red deer on carcass and meat characteristics, without the confounding factor of the level of stress at death. This is the first study to assess the influence of the four seasons of the year (September or autumn *vs*. January or winter *vs*. April or spring *vs.* June or beginning of summer) on meat quality of deer killed by stalking. It is unique because the population reduction stalking has been adapted in spring and summer to the seasonal needs of the study.

Hunting method also affects meat quality, especially through stress levels at death. In Spain, traditional driven hunts (Montería) often involve high stress, while stalking allows for low-stress harvesting. Previous studies have shown that ante mortem stress influences pH and other meat traits [12], but seasonal effects on fat and mineral composition appear to be independent of stress and more related to diet and antler physiology.

Therefore, the aim of this study was to evaluate how seasonality—assessed by hunting month—and the physiological effort of antler growth affect carcass traits and meat composition in adult wild male red deer harvested under minimal stress conditions. We hypothesized that seasonal changes in food availability and mineral mobilization during antlerogenesis would be associated with significant variation in meat quality parameters. Understanding the seasonal effects in meat quality and antler cycle mobilization of minerals may help to take decisions to balance hunting party or population density reduction with meat quality variations for its marketing.

## 2. Materials and methods

### 2.1. Study area and design

A total of 32 males of wild red deer of Iberian original genetic line (*Cervus elaphus*) were hunted on the same game estate (LM, Abenójar, Ciudad Real, south-central Spain: 38º53’N, 4º17’W) to avoid the variability caused by differences in plant diversity or productivity, or game management (e.g., supplying or not supplementary feed). The LM is a 900 ha fenced game estate with a population around 300 deer. The vegetation consists of natural pasture, shrub steppe, and Mediterranean forest, including as main species *Pistacia lentiscus*, *Pistacia terebinthus*, *Cistus ladanifer*, *Phillyrea angustifolia*, *Salvia rosmarinus*, *Quercus ilex*, *Erica spp.*, and *Genista spp.* Supplementary food high in protein and minerals is provided all year round with an estimate of 300–500 g per deer and day. The manufacturer is Piensos Hidalgo (Piedrabuena, Ciudad Real, Spain) and its composition is shown on Table 1).

**Table 1:** Feed Composition of supplement offered to wild deer.

Nutrient	Value	Unit
Crude protein	16.70	%
Crude fiber	18.71	%
Crude fat	2.63	%
Crude ash	5.45	%
**Vitamin or mineral**		
Vitamin A (3a672a)	10,000	UI/kg
Vitamin D3 (3a671)	1,000	UI/kg
Vitamin E (3a700)	45	mg/kg
Calcium	0.85	%
Sodium	0.29	%
Iron carbonate (E1)	50	mg/kg
Cobalt carbonate monohydrate (3b302)	0.15	mg/kg
Manganese oxide (3b502)	20	mg/kg
Zinc oxide (3b603)	38	mg/kg
Zinc chelate of glycine hydrate (3b607)	0.90	mg/kg
Copper sulfate pentahydrate (3b401)	9	mg/kg
Selenium sodium selenite (3b801)	0.12	mg/kg
Selenium Selenium-enriched yeast (3b82)	2.00	mg/kg

Deer were distributed in four different groups according to hunting period. The first group of deer were hunted in September 2020 (autumn, *n* = 8). The second one was hunted in January 2021 (winter, *n* = 8). The third group was hunted in April 2021 (spring, *n* = 8), coinciding with the onset of osteoporosis. Finally, the fourth group was hunted in June 2021 (beginning of summer, *n* = 8), coinciding with the middle of the period of osteoporosis in males. The game estate has a continental Mediterranean climate characterized by hot, dry summers and cold winters. Mean annual precipitation is low (~400–450 mm), with rainfall concentrated in autumn and spring and a pronounced summer drought. The average temperature registered was 21.4, 6.5, 14.0, and 23.3 ºC for September 2020, January 2021, April 2021, and June 2021, respectively and the average rainfall was 30.9, 63.3, 67.8, and 17.3 L/m^2^ for September 2020, January 2021, April 2021, and June 2021, respectively (reference weather station Abenójar).

Shots entry and exit wounds were in the cranial thoracic region. All samples were obtained by stalking (sudden low-stress death that did not involve a chase by dogs).

### 2.2. Carcass quality traits

The BW at slaughter was recorded for each experimental animal. Animals were exsanguinated, eviscerated, and decapitated at the atlanto-occipital junction in the countryside. Carcass weight was recorded for each experimental animal and data were used to calculate carcass yield.

The animals were shot either in hunting parties, or to reduce the population density as a form of game management. In the latter case, the game estate owner and coauthor (YF), adapted the date of shooting the animals to our requirements. Also, adult males age was shot aiming to exclude yearlings and animals of 2 years based on a long experience (although, at the several hundred metres where the hunter was, is rather difficult), but precise age could only be assessed after death. Only in the hunting party of September (which was not for population reduction, but sport hunting) a large number of animals was present, and we selected males that looked more clearly adults. In each carcass, age was determined by three independent trained wildlife veterinarians (different from the authors of the current paper) who followed guidelines reported by Brown and Chapman [14], using tooth eruption evaluation, wear patterns, and wear score of mandibular molars. In 84% of the carcasses, the estimated age was the same for the three evaluators. In the other carcasses the estimates were slightly different, and the age assigned was the arithmetic mean between the values assessed by the experts as indicated by Lorenzo et al. [1]. The age of animals according to month were: January, 2.9 ± 0.7 years; April 4.5 ± 1.2 years; June, 2.8 ± 0.5 years; September, 8.4 ± 2.8 years. Unfortunately, shooting depends on the opportunity of an animal appearing on view of the hunter, age cannot be easily determined by sight, and thus, it cannot be controlled easily as in a farm.

### 2.3. Meat quality traits

From each carcass, one of us (YF) sampled the LTL muscle, and the meat samples were vacuum packed and transported to the laboratory (Centro Tecnolóxico da Carne, Ourense, Spain) under refrigerated conditions via express delivery (before 10 a.m. the following day). This means that the time elapsed between death and laboratory analysis was less than 24h, and most of the time the samples were stored at about 5ºC.

Each LTL sample was divided into six steaks. The first three steaks were used to determine pH, colour, and chemical composition, respectively. The fourth and fifth steaks were used to determine the cooking losses and the shear force, respectively, whereas the sixth steak was used for the analysis of FA and mineral content. The external fat was removed from each sample and meat was minced and mixed to produce a homogeneous mixture before samples were submitted to chemical analysis.

Intramuscular pH was recorded at 72 h after hunting on the LTL. A digital portable meat pH-meter (Hanna Instruments, Eibar, Spain) with a glass electrode shaped to easily penetrate meat was used for the measurements. At the beginning, the pH meter was calibrated using solutions with pH values of 4 and 7 (Crison Instruments, Lainate, Italy and Barcelona, Spain) and it was also automatically calibrated for muscle temperature before each measurement as described by Vargas-Ramella et al. [15]. In addition, the incidence of DFD meats (pH at 72 h *post-mortem* above 6) was calculated.

For colour measurements, LTL samples were allowed to bloom directly in contact with air for 1 h. Objective measures of meat colour [16] including lightness (a greater L* value is indicative of a lighter colour), redness (a greater a* value is indicative of a redder colour), and yellowness (a greater b* value is indicative of a more yellow colour) were determined using a portable colorimeter (Konica Minolta CM-600d, Osaka, Japan) with a pulsed xenon arc lamp filtered to illuminate D65 lighting conditions, 0° viewing angle geometry, and 8 mm aperture size. Before each series of measurements, the colorimeter was calibrated with a white ceramic tile according to manufacturer recommendations. Three measurements were performed on each sample by rotating the detector system of 90° from the previous on three different points. Then, nine readings per sample were made at each point and averaged for statistical analysis, as described by De Palo et al. [17]. Additionally, chroma (C*) and hue angle (H°) were calculated as C* = (a*^2^ + b*^2^)^1/2^ and H° = arc tan (b*/a*), respectively [18].

Moisture, crude protein, and crude ashes of LTL were assessed according to the International Organization for Standardization recommended standards [19–21] while IMF was extracted and quantified according to the American of Oil Chemists´s Society Official Procedure Am 5–04 [22]. Briefly, moisture percentage was calculated by weight loss by the sample maintained in the oven (Memmert UFP 600, Schwabach, Germany) at 105°C until constant weight. Crude protein content was determined according to Kjeldahl total nitrogen method, multiplying the total nitrogen content by 6.25. Sample was subjected to reaction with sulphuric acid (cuprum sulphate was employed as a catalyst) in a digester (Gerhardt Kjeldatherm KB, Bonn, Germany). Organic nitrogen was transformed to ammonium sulphate, which was distilled in alkali conditions in a distillation apparatus (Gerhardt Vapodest 50 Carrousel, Bonn, Germany). Crude ashes percentage was calculated by weight loss experiment by maintaining the sample in a muffle furnace (Carbolite RWF 1200, Hope Valley, England) into a porcelain capsule at 600°C until constant weight. For IMF content determination, samples were subjected to a liquid-solid extraction using petroleum ether 40–60ºC in an extractor apparatus (AnkomHCI Hydrolysis System, Macedon NY, USA) at 90°C during 60 min. The IMF content was obtained based on gravimetric difference. The content of moisture and crude protein in the evaluated muscle of red deer was used to calculate the W/P as indicated by Milczarek et al. [23]. Energy value was calculated using individual energy factors for protein (4.00 kcal/g, 16.78 kJ/g) and fat (9.00 kcal/g, 37.62 kJ/g) [24].

Cooking losses of LTL was calculated as described by Pateiro et al. [25] (Cooking loss = (raw meat weight – coocked meat weight/raw meat weight) x 100). Briefly, steaks were cooked placing vacuum package bags in a water bath with automatic temperature control (JP Selecta, Precisdg, Barcelona, Spain) until they reached an internal temperature of 70°C, controlled by thermocouples type K (Comark, PK23M, UK), connected to a data logger (Comark Dilligence EVG, N3014, UK). After cooking, samples were cooled in a circulatory water bath set at 18°C for 30 min and the percentage of cooking loss was calculated. The Warner Bratzler shear force was analyzed as described by Lorenzo and Carballo [26]. All samples were cut perpendicular to the muscle fiber direction at a crosshead speed of 3.33 mm/min. A texture analyzer (TA-XT2, Stable Micro Systems, Godalming, UK) was used. Seven pieces of meat of 1 × 1 × 2.5 cm (height × width × length) were removed parallel to the muscle fiber direction. Samples were completely cut using a Warner Bratzler shear blade with a triangular slot cutting edge (1 mm thickness). Maximum shear force, shown by the higher peak of the force time curve, represents the maximum resistance of the sample to the cut.

### 2.4. Mineral content

For mineral determination, ashes previously obtained were dissolved in 10 mL of 1 M HNO_3_. The mineral elements (Ca, Fe, K, Mg, Na, P, Zn, Cu) were quantified by inductively coupled plasma (ICP) optical emission spectrometry, according to the procedure described by Lorenzo et al. [27] using a Thermo-Fisher ICAP 6000 plasma emission spectrometer (Thermo-Fisher, Cambridge, UK), equipped with a radio frequency source of 27.12 MHz, a peristaltic pump, a spraying chamber, and a concentric spray nebulizer. The system was totally controlled by ICP software using 99.996% liquid argon plasma gas (Praxair, Madrid, Spain). The final value for the content of each element was calculated as the average of three determinations for each sample.

### 2.5. FA methyl ester content

For the analysis of FA methyl esters, total fat was extracted from 10 g of ground LTL sample [28]. Fifty milligrams of fat were used to determine the FA profile. Total FA were quantified according to Domínguez et al. [29]. For the FA transesteriﬁcation, 4 mL of a sodium methoxide (2%) solution was added to the fat samples, vortexed every 5 min during the 15 min at room temperature, then 4 mL of a H_2_SO_4_ solution (in methanol at 33%) was added, vortexed for a few seconds, and vortexed again before adding 2 mL of distilled water. The organic phase (containing FA methyl esters) was extracted with 2.5 mL of hexane. Separation and quantification of the methyl esters was carried out using a gas chromatograph (GC-Agilent 7890B; Agilent Technologies Spain, S.L., Madrid, Spain), equipped with a ﬂame ionization detector and an automatic sample injector HP 7683, and using a Supelco SPTM-2560 fused silica capillary column (100 m, 0.25 mm internal diameter, 0.2 μm ﬁlm thickness; Supelco Inc., Bellafonte, PA, USA), following the chromatographic conditions described by Domínguez et al. [29]. Individual FA methyl esters were identiﬁed by comparing their retention times with those of authenticated standards. Data were used to calculate the total content of SFA, MUFA, and PUFA, PUFA/SFA ratio, total content of *n*-6 and *n*-3 and their ratio, and long chain *n*-3 PUFA. Additionally, lipid quality indices were calculated, i.e., NV according to Estévez et al. [30] and h/H according to Santos-Silva et al. [31]. Finally, IA and IT were calculated according to Ulbricht and Southgate [32]. Data are showed as mg/100 g of muscle and as g FA/100 g of total FA.

### 2.6. Ethics statement

The animals in this study were deer living in a game estate in conditions similar to those free-ranging (the state has 900 ha). They were shot by population regulation purposes, with the date of shooting adapted to the study. However, the numbers are included within the management program of the game estate to keep the population stable and avoid overpopulation. The shooting was performed by one of the authors, YF, who is a hunter and manager of the game estate, and has all legal permits for this procedure. The slaughter of the hunted animals is regulated by the Regional Hunting Law of Castilla la Mancha [33], and no ethical committee approval is necessary for this study.

### 2.7. Statistical analysis of the data

Normality was assessed using the Shapiro–Wilk test, and homogeneity of variances using Levene’s test. Data were normally distributed with homocedasticity. A general linear model test was performed to study the effects of hunting month (fixed effect) on carcass and meat quality characteristics, and nutrition value traits (dependent variables). To discriminate which groups differ from others, as shown on tables, a Tukey test was performed. In all cases, the experimental unit was the sample excised from each individual animal (*n* = 8). Differences were considered significant at p < 0.05. Values are given as means and standard error of mean. All analyses were carried out with SPSS version 22.0 (2013) (SPSS Inc., NY, USA).

## 3. Results

### 3.1. Culling body weight (BW) and carcass quality traits

Deer hunted in April were the heaviest compared to deer hunted in January, with deer hunted in September and June having intermediate values (p = 0.004. Table 2). However, the highest carcass yield was observed for deer hunted in September compared to deer hunted in January and June, with deer hunted in April being intermediate (*p* < 0.001).

**Table 2:** Effect of hunting month on slaughter body weight (BW), carcass yield, and meat quality traits of adult red deer males.

Items	Hunting month				SEM^1^	*p*-Value
	September	January	April	June		
Slaughter BW, kg	142.6^ab^	114.0^c^	150.8^a^	122.6^bc^	4.42	0.004
Carcass weight, kg	96.6^a^	67.7^c^	93.8^b^	72.4^c^	3.62	<0.001
Carcass yield, %	67.3^a^	59.2^c^	62.2^b^	59.0^c^	0.84	<0.001
pH_72_	5.68^a^	5.63^a^	5.51^b^	5.54^b^	0.019	0.002
Colour traits						
Lightness (L*)	32.4	32.7	34.7	34.1	0.38	–
Redness (a*)	15.5	15.9	16.6	14.0	0.39	–
Yellowness (b*)	10.7	10.8	12.1	10.1	0.33	–
Chroma (C*)	18.8	12.2	20.5	17.3	0.51	–
Hue angle (H rad)	0.60^ab^	0.59^b^	0.63^a^	0.62^a^	0.00497	0.026
Chemical composition, %						
Moisture	75.3	75.5	75.2	74.7	0.13	–
Crude protein	22.3	22.3	22.8	23.1	0.13	–
Intamuscular fat	1.23^a^	1.06^ab^	0.83^b^	1.03^ab^	0.045	0.016
Crude ashes	1.18	1.18	1.19	1.17	0.01	–
Water protein ratio	3.38	3.39	3.31	3.24	0.0254	–
Energy value, kJ/100 g	420.35	413.93	413.07	426.17	2.420	–
Cooking losses, %	26.4	24.0	25.4	26.0	0.53	–
Shear force, N/cm^2^	44.5	42.5	30.1	47.2	38.5	–

Different superscript letters within a row denote significant differences (p ≤ 0.05). ^1^ The experimental unit was the individual male (*n* = 8).

### 3.2. Meat quality traits

Concerning physicochemical parameters (Table 2), pH was higher for September and January carcasses than for April and June ones (p = 0.002). The pH_72_ did not exceed 6 (dark, firm, and dry (DFD) meats) in any case. As shown on Table 2, hunting period did not affect lightness, redness, yellowness, or chroma, although it did affect hue angle, indicating that the overall colour intensity did not change, but the colour nuance (i.e., the angle defined by a* and b*) shifted slightly. Meat composition was affected by season. Thus, loins from deer hunted in April and June tended to have a higher content of crude protein than those from deer hunted in September and January (p = 0.097). Also, loins from deer hunted in September had higher contents of IMF than loins from deer hunted in April, with loins from deer hunted in January and in June being intermediate (p = 0.016). However, hunting season did not affect moisture and ashes content of meat neither water–protein ratio (W/P) nor energy value.

Cooking losses were not affected by hunting period, and neither was shear force.

### 3.3. Mineral content

Potassium (K) was the most abundant micromineral in meat (42.8% of total, Table 3), followed by P (35.6%) and Na (13.7%). Month influenced on the *Longissimus thoracis et lumborum* (LTL) contents of most minerals analysed. In fact, meat samples collected in April had the highest content of Ca (p < 0.001), K (p = 0.027), and Mg (p < 0.001). The Fe content was higher in meat from deer hunted in September and January compared to that from deer hunted in April and June (p = 0.001). Meat from deer hunted in September had the lowest content of Na compared to meat from deer hunted in January, April, and June (p < 0.001). The highest P content was observed for January and April meats comparing to June meat, with September meat being intermediate (p = 0.006). The meat with the highest content of Zn was that obtained in January (p < 0.001). Also, the Cu content of meat tented (p = 0.069) to be higher for meat from deer hunted in January than for meat from deer hunted in the other months.

**Table 3:** Effect of hunting month on mineral content (mg/100 g of muscle) of meat from adult males of red deer.

Mineral	Hunting month				SEM^1^	*p*-Value
	September	January	April	June		
Ca	5.9^b^	7.8^b^	98.5^a^	8.4^b^	8.40	<0.001
Fe	3.5^a^	3.8^a^	2.9^b^	2.6^b^	0.134	0.001
K	248.8^b^	273.8^b^	314.4^a^	284.1^b^	4.91	0.027
Mg	21.0^c^	25.3^b^	33.5^a^	27.1^b^	1.07	<0.001
Na	62.7^b^	106.3^a^	102.9^a^	108.3^a^	4.35	<0.001
P	234.2^ab^	259.4^a^	252.7^a^	206.1^b^	5.98	0.006
Zn	2.92^b^	4.10^a^	2.37^b^	2.28^b^	0.199	<0.001
Cu	0.14	0.17	0.14	0.15	0.005	0.069

Different superscript letters within a row denote significant differences (p ≤ 0.05). ^1^ The experimental unit was the individual male (*n* = 8).

### 3.4. Fatty Acid methyl ester content

The main fatty acids (FA) were saturated FA (SFA, around 39.6% of total FA. Table 4), followed by monounsaturated (MUFA, around 32.7% of total FA), and polyunsaturated (PUFA, around 27.8% of total FA), which showed some differences among hunting months as shown on each table. Meat from deer hunted in summer (June) had contents of SFA, MUFA, PUFA, total *n*-6, total *n*-3, long chain *n*-3 PUFA, and nutritional value (NV) higher than meat from deer hunted at the other months (p < 0.001. Table 5). On the other hand, meat from deer hunted in January had the highest PUFA/SFA ratio, *n*-6/*n*-3 ratio, and hypocholesterolemic/hypercholesterolemic ratio (h/H) (p < 0.001). Deer hunted in April and June had the highest index of atherogenicity (IA) (p < 0.001), while deer hunted in January had the lowest index of thrombogenicity (IT) (p < 0.001).

**Table 4:** Effect of hunting month on saturated (SFA), monounsaturated (MUFA), and polyunsaturated (PUFA) fatty acid (FA) profile (mg/100 g of muscle) of meat from adult red deer males.

Fatty acid	Hunting month				SEM^1^	*p*-Value
	September	January	April	June		
**SFA**						
C14:0	39.74^b^	5.01^c^	59.74^a^	65.20^a^	5.668	<0.001
C15:0	4.07^b^	2.52^c^	4.80^b^	5.67^a^	0.264	<0.001
C16:0	194.4^c^	63.3^d^	285.2^b^	387.8^a^	25.83	<0.001
C17:0	2.84^c^	1.95^c^	4.38^b^	5.97^a^	0.331	<0.001
C18:0	77.2^b^	70.6^b^	78.6^b^	114.8^a^	4.39	<0.0014
Total SFA	321.1^c^	144.9^d^	443.4^b^	591.6^a^	36.05	<0.001
**MUFA**						
C14:1*n*-5	12.63^b^	1.17^c^	23.63^a^	26.61^a^	2.205	<0.001
C15:1*n*-5	59.9^a^	52.1^ab^	44.5^b^	45.2^b^	1.85	0.004
C16:1*n*-7	65.31^b^	7.72^c^	87.10^b^	120.40^a^	9.135	<0.001
C 18:1-*11t*	0.56^c^	0.38^c^	4.94^b^	6.11^a^	0.504	<0.001
C18:1*n*-7	35.8^a^	10.0^c^	24.1^b^	33.2^ab^	2.60	<0.001
C18:1*n*-*9*	105.6^bc^	74.6^c^	119.2^b^	160.4^a^	7.85	<0.001
Total MUFA	281.6^b^	147.2^c^	308.5^b^	395.9^a^	21.10	<0.001
**PUFA**						
C18:2*n*-6	65.2^c^	98.4^ab^	89.4^b^	110.6^a^	4.45	<0.001
C18:3*n*-3	11.9^c^	17.6^b^	32.9^a^	32.6^a^	1.99	<0.001
C20:3*n*-6	7.01	6.25	5.34	6.17	0.246	–
C20:4*n*-6	41.6^ab^	46.4^a^	35.8^b^	37.4^b^	1.36	0.018
C20:5*n*-3 (EPA)	10.8^b^	10.9^b^	12.1^b^	15.9^a^	0.505	<0.001
C22:5*n*-3 (DPA)	21.7^bc^	19.5^c^	24.2^b^	30.5^a^	1.00	<0.001
C22:6n-3 (DHA)	2.63^b^	2.75^ab^	2.29^b^	3.68^a^	0.181	0.085
Total PUFA	163.3^c^	204.0^b^	212.0^b^	248.7^a^	7.44	<0.001

Different superscript letters within a row denote significant differences (p ≤ 0.05). ^1^ The experimental unit was the individual male (*n* = 8).

**Table 5:** Effect of hunting month on fatty acid (FA) profile (mg/100 g of muscle) indexes of meat from adult red deer males.

Items	Hunting month				SEM^1^	*p*-Value
	September	January	April	June		
Total FA	766^b^	496^c^	964^b^	1.236^a^	60.0	<0.001
PUFA/SFA^2^	0.55^b^	1.45^a^	0.51^b^	0.42^b^	0.095	<0.001
∑*n*-6	114.5^c^	152.0^ab^	132.5^bc^	156.8^a^	4.83	<0.001
∑*n*-3	47.8^c^	51.3^c^	73.3^b^	84.5^a^	3.39	<0.001
∑*n*-6/∑*n*-3	2.41^b^	2.97^a^	1.83^c^	1.89^c^	0.110	<0.001
Long chain *n*-3 PUFA^3^	35.2^b^	33.1^b^	38.6^b^	50.1^a^	1.54	<0.001
Nutritional value^4^	1.36^b^	0.39^c^	1.64^ab^	1.72^a^	0.120	<0.001
h/H^5^	1.24^b^	3.83^a^	0.94^b^	0.84^b^	0.269	<0.001
IA^6^	0.78^b^	0.23^c^	1.00^a^	1.02^a^	0.073	<0.001
IT^7^	0.90^a^	0.45^b^	0.99^a^	1.08^a^	0.056	<0.001

Different superscript letters within a row denote significant differences (*p* ≤ 0.05).

^1^The experimental unit was the individual male (*n* = 8).

^2^Polyunsaturated fatty acid (PUFA)/saturated fatty acid (SFA).

^3^C20:5*n*-3 + C22:5*n*-3 + C22:6*n*-3.

^4^Σ(C12:0 + C14:0 + C16:0)/Σ(C18:1*n*‒9 + C18:2*n*‒6).

^5^Hypocholesterolemic/hypercholesterolemic ratio = [Σ(C18:1*n*‒7 + C18:1*n*‒9 + C18:2*n*‒6 + C18:3*n*‒3 + C20:3*n*‒6 + C20:4*n*‒6)/Σ(C14:0 + C16:0)]. Ratio h/H calculated according to Santos-Silva et al. [31].

^6^Index of atherogenicity = [C12:0 + 4*C14:0 + C16:0]/[(∑MUFA)+(∑PUFA)], with MUFA being the monounsaturated fatty acid. IA and IT were calculated according to Ulbricht and Southgate [32].

^7^Index of thrombogenicity = [C14:0 + C16:0 + C18:0]/[(0.5*∑MUFA)+(0.5*∑*n*‒6)+(3*∑*n*‒3)+(∑*n*‒3)/∑*n*‒6)], with MUFA being the monounsaturated fatty acid.

Regarding SFA, the dominant FA were palmitic (C16:0) and stearic (C18:0) acids with mean values around 23.2 and 10.8% of total FA, respectively followed by myristic acid (C14:0, 3.99% of total FA). These FA varied with hunting month with the highest values being, in general, for samples collected in June (p < 0.001). Concerning to MUFA, oleic acid (C18:1*n*-9) was the dominant FA with the 13.5% of total FA, showing differences among seasons (p < 0.001). Trans-vaccenic acid (TVA; C18:1-11t), an important precursor of conjugated linoleic acid, differed among collection periods being the highest value for deer hunted in June (p < 0.001). Among PUFA, the linoleic acid (C18:2*n*-6) was the main FA (88.8 mg/100 g of muscle as average and 12.4% of total FA) followed by arachidonic acid (C20:4*n*-6, 41.2 mg/100 g of muscle as average and 6.0% of total FA), with both being affected by season (p < 0.05). The α-linoleic acid (C18:3*n*-3) and docosapentaenoic acid (C20:5*n*-3, EPA) displayed differences among seasons, presenting the highest values in meat samples from animals hunted in April and June for C18:3*n*-3 and in June for EPA (p < 0.001). In general, similar results were observed when data were showed as g/100 g of FA (Tables 5–7).

**Table 6:** Effect of hunting month on saturated (SFA), monounsaturated (MUFA), and polyunsaturated (PUFA) fatty acid profile (g/100 g of fatty acid; i.e., on a percentage basis) of meat from adult red deer males.

Fatty acids	Hunting month				SEM^1^	*p*-Value
	September	January	April	June		
SFA						
C14:0	5.02^a^	0.93^b^	5.87^a^	5.40^a^	0.461	<0.001
C15:0	0.53	0.51	0.49	0.51	0.0090	–
C16:0	24.7^b^	12.7^c^	28.5^ab^	29.2^a^	1.46	<0.001
C17:0	0.37^b^	0.40^b^	0.45^ab^	0.49^a^	0.014	0.005
C18:0	10.3^b^	7.1^a^	8.0^c^	9.5^bc^	0.51	<0.001
Total SFA	41.3^a^	29.0^b^	44.4^a^	46.1^a^	1.53	<0.001
MUFA						
C14:1*n*-5	1.57^b^	0.21^c^	2.62^a^	2.23^b^	0.205	<0.001
C15:1*n*-5	8.04^b^	10.6^a^	4.67^c^	3.86^c^	0.572	<0.001
C16:1*n*-7	8.24^a^	1.48^b^	10.25^a^	9.96^a^	0.782	<0.001
C18:1-*9t*	0.11^b^	0.14^b^	0.21^a^	0.19^a^	0.00894	<0.001
C18:1-*11t*	0.067^b^	0.078^b^	0.57^a^	0.51^a^	0.0466	<0.001
C18:1*n*-*9*	13.8	14.8	12.1	13.2	0.36	0.056
Total MUFA	36.6^a^	29.4^c^	33.4^ab^	32.8^bc^	0.70	<0.001
PUFA						
C18:2*n*-6	8.7^b^	20.0^a^	9.3^b^	9.4^b^	1.10	<0.001
C18:3*n*-3	1.58^c^	3.62^a^	3.41^ab^	2.72^b^	0.200	<0.001
C20:3*n*-6	0.97^ab^	1.28^a^	0.56^bc^	0.52^c^	0.0791	<0.001
C20:4*n*-6	5.68^b^	9.43^a^	3.81^bc^	3.24^c^	0.549	<0.001
C20:5*n*-3 (EPA)	1.63^ab^	2.26^a^	1.26^b^	1.35^b^	0.124	0.008
C22:5*n*-3 (DPA)	2.97^b^	4.00^a^	2.48^b^	2.57^b^	0.166	<0.001
C22:6*n*-3 (DHA)	0.36^ab^	0.55^a^	0.29^b^	0.31^b^	0.0331	0.010
Total PUFA	22.2^b^	41.6^a^	22.2^b^	21.1^b^	2.00	<0.001

Different superscript letters within a row denote significant differences (*p* ≤ 0.05). ^1^ The experimental unit was the individual male (*n* = 8).

**Table 7:** Effect of hunting month on indexes of fatty acid (FA) profile (g/100 g of fatty acid; i.e., on a percentage basis of Table 6) in meat from adult red deer males. Highest values in bold.

Items	Hunting month				SEM^1^	*p*-Value
	September	January	April	June		
PUFA/SFA^2^	0.56^b^	1.45^a^	0.51^b^	0.46^b^	0.0942	<0.001
∑*n*-6	15.4^b^	**30.9** ^a^	13.9^b^	13.4^b^	1.64	<0.001
∑*n*-3	6.64^b^	**10.5** ^a^	7.63^b^	7.11^b^	0.449	<0.001
∑*n*-6/∑*n*-3	2.38^b^	**2.97** ^a^	1.83^c^	1.89^bc^	0.111	<0.001
Long chain *n*-3 PUFA^3^	4.96^ab^	6.80^a^	4.03^b^	4.24^b^	0.309	<0.001
Nutritional value^4^	1.36^a^	0.39^b^	**1.64** ^a^	1.55^a^	0.113	<0.001
h/H^5^	1.24^b^	**3.83** ^a^	0.95^b^	0.93^b^	0.266	<0.001
IA^6^	0.78^a^	0.23^b^	0.95^a^	0.95^a^	0.068	<0.001
IT^7^	0.89^a^	0.45^b^	0.91^a^	0.99^a^	0.052	<0.001

Different superscript letters within a row denote significant differences (*p* ≤ 0.05) in a Tukey test.

^1^The experimental unit was the individual male (*n* = 8).

^2^Polyunsaturated fatty acid (PUFA)/saturated fatty acid (SFA).

^3^C20:5*n*-3 + C22:5*n*-3 + C22:6*n*-3.

^4^Σ(C12:0 + C14:0 + C16:0)/Σ(C18:1*n*‒9 + C18:2*n*‒6). Higher value, less favourable. Lowest value in January.

^5^Hypocholesterolemic/hypercholesterolemic ratio = [Σ(C18:1*n*‒7 + C18:1*n*‒9 + C18:2*n*‒6 + C18:3*n*‒3 + C20:3*n*‒6 + C20:4*n*‒6)/Σ(C14:0 + C16:0)]. Higher h/H = more healthy.

^6^Index of atherogenicity = [C12:0 + 4*C14:0 + C16:0]/[(∑MUFA)+(∑PUFA)], with MUFA being the monounsaturated fatty acid. Lower IA = more healthy.

^7^Index of thrombogenicity = [C14:0 + C16:0 + C18:0]/[(0.5*∑MUFA)+(0.5*∑*n*‒6)+(3*∑*n*‒3)+(∑*n*‒3)/∑*n*‒6)], with MUFA being the monounsaturated fatty acid. Lower IT = more healthy.

## 4. Discussion

One of the reasons why consumers like game meat is its image of healthy product. Taste, nutritional value, and low-fat content are important quality parameters influencing their willingness to increase the future game meat consumption [8]. However, the difficulty for a regular supply of game meat because of its seasonality (hunting season is restricted to some months) is a significant barrier to its consumption [34], which for deer has been filled by the farmed deer meat, mainly from New Zealand [35].

Although most of meat from Spain is produced in stressful driven hunts (known as “*Montería*”) during autumn and winter, selective stalking (low-level stress death) in Spain and other countries during summer yields a second source of meat in through culling poor trophy animals or to reduce population density [12]. Thus, inevitably, the comparison between the most common marketed deer meats shows a mixed effect of hunting type with seasonality. Most of the literature review focusing on quality of venison shows that the animals studied (farm raised or wild deer) were hunted during one particular season of the year, being either the autumn or the winter.

Despite wild red deer is the more common source of deer meat production, available information about the influence of hunting season on carcass and meat quality traits and nutrition value is very scarce. In addition, the information available about meat quality of deer hunted by stalking is even more scarce. Serrano et al. [12], compared the quality of main types of hunted red deer meat obtained in Spain (stressful-winter *vs*. stalking-summer) compared to farmed venison from New Zealand. However, these authors studied the most commonly available deer meat: from deer hunted in winter by stressful chase, and in summer by stress-free stalking, thus producing a mixed effect that needed to be clarified. For this reason, we designed the present study to isolate season effects by culling animals always stress-free by stalking in all months.

### 4.1. Slaughter body weight and carcass quality traits

Wild deer are typically found in areas with marked seasonal variation in climate and food availability [36]. This, together with the polygynous reproduction of the deer, which means that males compete heavily for a harem of females, leaves a number of males without the possibility of reproduction. As a consequence, male deer have evolved a highly seasonal pattern of growth. This pattern has a maximum accretion of body tissue (muscle and fat) in spring and summer (period of high plant productivity and when, in addition, antler grows), and minimal accretion, or even loss of body mass, during autumn and winter, when males do not feed because they are fighting or guarding females of their harem (despite food is highly available also in autumn) [36,37]. This interpretation is supported by the finding of a reduction of rumen volume by about one third during winter in red deer [38]. This seasonal variation in protein accretion and catabolism could influence meat tenderness and other meat quality attributes such as water holding capacity [14].

Month of culling influences growth [5], carcass traits [10], and meat quality of cervids [9], because there is a difference in the body condition of wild animals related to availability of food in the habitat, and the amount of body fat, and resources spent in autumn and winter.

In the current trial, deer hunted in September had the heaviest carcasses compared to deer hunted in January, April, and June. The influence of season on carcass weight has been previously observed [7,37,39]. The information provided by carcass weight is very important due to its potential as a bioindicator of body mass in order to infer physiological states and nutritional conditions [40].

The lowest slaughter body weight (BW), carcass weight, and carcass yield observed in January in the current study is consistent with the effect that deer did not feed during autumn because they devoted time and energy to mate and guard females, as well as to fight males. Another factor explaining this may also be the hardship of winter with low availability of food and the use of fat and body resources. It is more difficult to explain why in June, after the recovery of BW during spring and with greater food availability, the slaughter BW, carcass weight, and yield are the second lowest. The reason maybe that in April the antler is just growing, but in June it is rather well developed, and it shows what type of trophy will be when it is fully grown and cleaned of skin in late August. In this situation, a hunter in a game estate will avoid killing animals that show potentially big trophies, so that, inadvertently, they may have killed adults of poorer trophy and younger (in fact, September males were older 8.4 ± 2.8 y than those in June 2.8 ± 0.5 y). As is already known from scientific papers as early as XIX century that antler weight is related to BW (reviewed in Landete-Castillejos et al. [11]) this would have influenced the BW and related parameters of the animals selected in June.

García del Rincón-Garoz et al. [41], found that males had lower carcass weight in winter than in autumn, which agrees with current results. Also, these authors observed a lower carcass yield in winter than in autumn, also agree with current results and with those observed by Pérez-Serrano et al. [10]. However, in García del Rincón-Garoz et al. [41],(2023) study males and females were within one group. Red deer, like other temperate ruminants, survive winters by metabolically pre-programming themselves to undergo a negative energy balance through decreased metabolism and loss of body fat reserves [42]. Thus, the thermoregulatory stress and limitations in forage access and availability leads to fluctuations in carcass weight [43]. Therefore, these results might be due to the inverse relationship between body size and the relative rate of metabolic requirement, resulting in weight loss [44]. However, it would be difficult to explain a similar carcass yield in June by any hypothesis that it is not an inadvertent bias of the hunter to kill males of lower trophy (therefore, lower BW and condition). It should be remembered that deer in April have just cast the antlers, and in June they are in the last half of its antler growth, so that animals can be chosen not to kill to reduce population density by antler size, rather than using body weight (which is long known to be related to antler size although genetics and food availability also influence it -reviewed in Landete-Castillejos et al. [11]).

### 4.2. Meat quality traits

In principle, a low stress death by stalking should result in a better quality of meat and, in fact, meat processing companies pay better prices for meat from stalked animals than for stress hunted meat, as evidenced by game estate owners and personal interviews [12]. Equally, meat from farmed deer reaches a higher price than that of wild ones because of its greater quality than stress hunted meat (the most common in the hunting season, as larger numbers of deer can be killed in a day of hunting party), and also because it is de-seasonalized [2].

Seasonal changes have been reported to have effects on meat quality of deer [9,11]. The final pH of deer meat analysed in the current study indicates a high quality of the product. In fact, no cases were detected for DFD percentage meat (final pH values above 6). There were, however, seasonal differences in pH: autumn and winter carcasses had values of pH (pH72) higher than those in spring and summer carcasses. Pérez-Serrano et al. [10], also did not find differences for pH between animals hunted in winter and autumn. As in the current study, Serrano et al. [12], found pH values in winter higher than those in summer, as Wiklund et al. [7] found in other conditions. Stress affects pH and thus, values in the present study by stalking were similar to stalking summer values but lower than stressful-chase in winter by Serrano et al. [12].

Meat IMF was affected by hunting period. Thus, loins from deer hunted in September had the highest IMF content, whereas it decreased in January, and further in April. This may be consistent with the fact that, at the end of the summer, the deer is in the best BW and condition (i.e., September), reflected in the greatest IMF of its meat. Then, throughout the autumn, the IMF is reduced as the male deer spend energy in reproduction and fighting, and because they do not feed. In winter, the level continues to decrease, and it is not surprising that at the onset of spring (21^st^ of March, but our closest date is April) the recovery cannot reach the levels at the end of the summer early autumn (September). The low IMF observed in June, despite it is at the end of the spring early summer (lower than in January) may again point to a bias of the hunter towards poorer trophies. Therefore, lower BW may have biased the sample towards a lower body condition, and lower IMF. Previously, Pérez-Serrano et al. [10], found that loins from deer hunted in autumn had higher contents of IMF than loins from deer hunted in winter, which agrees with current results. The fat content of meat is very important for its quality because it confers juiciness, texture, and flavour, especially during heat treatment or cooking. Thus, meat from deer hunted in autumn could be more desirable than that from deer hunted in winter [10] and in the other seasons, as reported in the current study.

As an average, the shear force observed in the current study was 40.6 N, a value higher than the 30.2 N observed by Cawthorn et al. [45], for the LTL muscle from male wild fallow deer, and lower than the 22.1 (stags) and 19.2 (hinds) N observed by Piaskowska et al. [46] for the *Longissimus lumborum* muscle from wild fallow deer and to the 19.2 N observed by Maggiolino et al. [47]. In the current study, shear force was not affected by month, as in Pérez-Serrano et al. [10] who only observed a non-significant trend tending to be higher for winter than for autumn loins. The cause of the differences among authors are unknown but variations in muscle tenderness at slaughter and during post mortem storage could be the result of various interrelated factors, including pH, amount of connective tissue, IMF content, proteolytic enzyme activity and age of the animal [45].

### 4.3. Mineral content

Mineral composition presented in deer meat is closely related to the natural environment as they graze and browse [48]. In fact, consumption of complex pastures often results in higher concentrations of vitamins and minerals in grass fed animals [49]. Regarding macrominerals, as expected for muscle, K was the main one followed by P, and Na [10,12].

Changes observed for mineral composition of meat might be caused by the mobilization of skeleton minerals in support of antler growth as demonstrated by Serrano et al. [12]. Thus, the content of Ca in muscles was 10 times higher in April start of antler growth, than in January or September, and the peak of osteoporosis. The value of June similar to that of January may be explained by the start of recovery from skeleton osteoporosis. The study by Serrano et al. [12] compared winter vs. summer and found differences in values that differed by 30% (summer vs. winter), not by near to 1,200% as April with January in our study. In this sense, one-fragment contamination during sampling was ruled out. Although the seasonal difference is not so huge, the content of Mg, a mineral that can substitute Ca in bones or in the last stages of the growing antler [11], is again, higher in April and June (slightly lower than April) compared to September or January. Interestingly, this greater Mg content during antler growth was also found by Serrano et al. [12], comparing summer (June is our closest date) with winter. Another mineral showing cyclic osteoporosis effect is Zn, which is the mineral attached to Alkaline phosphatase, the enzyme depositing Ca on bones during mineralization [11]. The Zn content was 56% lower in April and June as compared to January (comparison average of April and June with the winter month). Similarly, Serrano et al. [12] found that Zn was 39% lower in summer than in winter. The explanation of this is that a great proportion of blood Zn is captured by the growing antler (and, in June, also by the bones recovering from osteoporosis), and less is deposited in the muscles. Iron was also lower in April and June than in September or January (25% lower). Serrano et al. [12] found also a content of Fe in summer 15% lower than in winter. This may be caused by the fact that Fe is used in catalase and other antioxidant enzymes [50], which are hardly needed in the growing antler because having the fastest tissue growth rate leads also potentially to the highest oxidative stress [13]. Surprisingly, P trends did not follow those of Ca or Mg, but the reason may lie in the other roles that P plays in the cellular metabolism apart from being part of the calcium phosphate in bones.

### 4.4. FA methyl ester content

Deer as other wild game meat contains low fat levels and a healthy FA composition [51–53], most likely because they are free ranging and the plant biodiversity constituting their diet [54,55]. The FA profile was influenced by season at least partly due to the fact that variations in forage eaten affect greatly FA composition [56]. In fact, such seasonal effects has been found even in FA profiles from farmed ruminants due to variations of pasture composition, ensiling and other factors [57,58].

Meat from deer hunted in summer had higher contents of SFA, MUFA, PUFA, n-6, n-3, long chain n-3 PUFA, and NV than in other seasons. In contrast, Serrano et al. [12] found that meat from deer hunted in autumn had higher contents of SFA and MUFA, and lower of PUFA than meat from deer hunted in winter. This is particularly interesting because both studies were conducted in the same game estate in a different year and probably reflect climate effect on plant diversity or productivity, leading to changes in diet composition even on the same place for different years.

The lower content of SFA on meat of deer hunting during winter might be due to lower intake of feed [59], and presumably, a diminished microbial fermentation at lower temperature [60]. This interpretation is supported by the finding of a reduction of rumen volume by about one third during winter in red deer [38]. Also, the lowest content of SFA on meat of deer hunting during winter might be due also to the lowest IMF content comparing to loins from deer hunted in September that had the highest IMF content [61].

Seasonal changes in FA composition caused by plant diversity can found be found even in farmed ruminants: Costa et al. [62] found that slaughter season influenced C18:2 cis-9 trans-11 (CLA) in veals, showing a higher content in autumn. Season also affected total MUFA, n-3 PUFA, and well as n-6/n-3 ratio.

Is there evidence for such change in fatty acid profile directly in pasture that can affect meat FA composition? There are at least two relevant studies. Krusinski et al. [63] reported in summer pasture, higher contents in most SFA, MUFA, and C18:2 n-6, which could explain our results. In addition, Meľuchová et al. [64] reported an increase in C16:0 and C18:2 n-6 from May to July compared to September. In general, fat from meat of wild animals has healthy PUFA/SFA and n-6/n-3 PUFAs ratios [52,53,63,65–67].

It is important to bear in mind the constraints of this study, some of which become specific strengths: 1) it is a single estate (but directly comparable in plant composition, climate, and management with previous study [12]); 2) the supplementation is common practice in game estates, but departs diet from one based on wild plants alone, and it is also impossible to control exact diet composition for an animal in the wild; 3) as animals are not picked up in cohorts in a farm, random shooting at distance led to some age structure effects by month.

## 5. Conclusion

Mineral composition displayed a clear seasonal pattern that appeared to be more closely associated with skeletal reserve mobilization during antler development than with changes in diet. In particular, Ca and Mg concentrations were elevated in April, whereas Fe and Zn levels declined in April and June, supporting the hypothesis of mineral mobilization linked to antler growth. Taken together, these results indicate that both seasonality and the physiological demands of antler development significantly influence meat quality and composition in wild male red deer, providing further insight into their nutritional ecology and the characteristics of game meat.
