# Supplementary material for: Effects of season and antler growth on carcass traits and meat quality in wild male red deer harvested by selective stalking
Source: PLoS One. 2026 Jul 8;21(7):e0352456. doi: 10.1371/journal.pone.0352456 (PMC13345254; doi:10.1371/journal.pone.0352456)
Supplement: Attachment — Submitted filename: Reviewer Report PONE-D-25-30036.pdf [file pone.0352456.s001.pdf]

Reviewer Report: PONE-D-25-30036
Manuscript Title: [**Effect of hunting month and physiological effort of antler growth on carcass and meat quality of wild red deer males killed stress-free**]
Journal: [**PLOS ONE**]

Reviewer Report

Summary of the Research and Overall Impression

The manuscript explores the influence of hunting month and the physiological demands of antler growth on carcass and meat quality traits in wild red deer. The topic is original and highly relevant to wildlife physiology, game meat science, and ecological management. The study addresses a novel research question that integrates seasonality with metabolic and mineral dynamics in deer.
Overall, the work is promising and potentially valuable, but several sections require clearer structuring, refinement of interpretation, and improved scientific precision to meet *PLOS ONE* standards.

Major Comments

(Lines 27–29) The sentence *"This study examines for first time the effects of seasonality…"* should be corrected for grammar (e.g., "for the first time") and revised for clarity. The authors should also state their specific hypotheses or objectives explicitly to strengthen the abstract's focus.

(Lines 25–39) The abstract currently blends background, results, and interpretation. A more structured flow would improve readability:
1–2 sentences for background/context;
1 sentence for the main aim;
2–3 sentences summarizing key findings with direction and significance;
1 concluding sentence emphasizing novelty or implications.

(Lines 31–34) While including *p*-values is good practice, ensure consistency (use "$p < 0.05$" uniformly). Mentioning the general type of statistical test (e.g., ANOVA or mixed model) would help readers appreciate the analytical rigor.

Interpretation vs. Data Presentation (Lines 35–38) Avoid causal phrasing such as “caused by.” Since this is observational research, use softer wording like “may be influenced by” or “is likely associated with” to align with descriptive study conventions.

Minor Comments

- Maintain consistent spacing around parentheses and symbols (e.g., $p < 0.05$).
- Replace “culled stress-free” with “harvested under minimal stress conditions” for more formal scientific tone.
- Clarify “loin muscle quality” by identifying the specific muscle studied (e.g., *Longissimus thoracis et lumborum*).
- Strengthen the concluding line by emphasizing novelty, e.g., *“These findings demonstrate that seasonal dynamics and physiological investment in antler growth jointly influence meat composition in wild red deer, offering new insights into nutritional ecology and game meat quality.”*

Introduction

(Lines 45–71) The introduction combines several themes—environmental sustainability, deer biology, antler physiology, and hunting practices—without a clear logical progression. Consider restructuring:

- **Paragraph 1:** Importance of venison as a sustainable protein source.
- **Paragraph 2:** Seasonal physiological effects on meat quality.
- **Paragraph 3:** Mineral mobilization and antler growth.
- **Paragraph 4:** Hunting context and stress effects.
- **Final paragraph:** Explicitly state study aims and hypotheses.

(Lines 82–88) The novelty and objectives should be emphasized more clearly to justify the study’s rationale.

(Lines 61–67) The discussion of “cyclic osteoporosis” is interesting but overly detailed for the Introduction. Condense this section and refer readers to the cited review ([11]) for further context.

(Lines 68–71) The statement that antler-derived biomolecules may have anti-aging or anti-cancer properties is speculative. It should be softened (e.g., “It has been suggested that…”) or omitted to maintain scientific objectivity.

Minor Notes

- Define abbreviations such as *FA* (fatty acids) upon first mention.

- Use the species name “red deer (*Cervus elaphus*)” consistently.
- Check reference formatting (use either “(1,2)” or “[1,2]” per journal style).
- Ensure that all numerical or quantitative claims are properly cited.

Results

(Lines 132–180) The observed seasonal variations are clearly presented, but biological interpretation should be strengthened. The authors should explicitly link observed trends (e.g., higher carcass yield in September) to nutritional status and antler growth demands, rather than presenting them descriptively.

(Lines 181–210) Mineral variation results (Ca, Mg, Fe, Zn) are intriguing but require deeper explanation. Discuss how these results relate to bone mobilization and antlerogenesis, citing relevant literature on mineral metabolism in cervids.

(Lines 211–250) The section on fatty acid profiles can be condensed. Focus on the biological implications of PUFA/SFA ratios and related health indices, rather than excessive numerical details.

- Ensure uniform significance notation ($p < 0.05$).
- Clarify if post-hoc corrections were applied for multiple testing.

Minor Suggestions Group related fatty acid indices or mineral results into summary tables to improve clarity and readability.

Discussion

(Lines 189–410) The Discussion is lengthy and partially repetitive. Consider condensing it by removing background information already presented in the Introduction. Each results subsection (carcass, meat traits, minerals, FA profile) is discussed in isolation; an integrative paragraph linking these findings through nutrition–antler growth–metabolism interactions would enhance coherence.Some statements (e.g., hunter bias, lines 244–250) are speculative and should be rephrased cautiously unless supported by data. Also, the Discussion relies heavily on the group’s own previous studies [10, 12]. A broader comparison with international studies would improve generalizability.

Minor Adjustments

- Simplify overly long sentences (e.g., lines 300–310).
- Standardize terminology (IMF, intramuscular fat, slaughter BW).

- End with a concise, practical statement highlighting management implications, e.g., *"Identifying the optimal hunting period could enhance both meat quality and resource sustainability."*
- **Materials and Methods**

(Lines 427–460) Although the design is balanced (n = 8 per month), age varies notably among months (~8 years in September vs. ~3 years in others). This age difference is a major confounder that could influence carcass and meat traits. Clarify whether age was statistically controlled (e.g., as a covariate), and if not, acknowledge this limitation explicitly.

(Lines 572–579) The General Linear Model includes only "hunting month" as a fixed factor. Given the complexity of biological variation, *age* and possibly *environmental factors* (temperature, rainfall) should be considered covariates. Indicate whether post-hoc tests were adjusted for multiple comparisons (e.g., Tukey HSD).

(Lines 463–466) Specify the time interval between death and muscle sampling, as this directly affects pH and biochemical parameters. Clarify whether carcasses were cooled before sampling or analyzed fresh.

Minor Comments

- Use decimal points consistently (e.g., 2.9 ± 0.7 years).
- Correct typographical error: "cocked meat weight" → "cooked meat weight."
- Ensure full citation details for AOAC and ISO references (e.g., publication year, method number).

Overall Evaluation

The manuscript presents a valuable contribution to the understanding of seasonal physiology and meat quality in wild red deer. However, revisions are needed to improve structure, refine interpretation, and strengthen statistical transparency. Addressing the confounding effect of age, clarifying methodological details, and condensing the Discussion will substantially enhance the study's clarity and scientific impact.
