# Supplementary material for: Effects of season and antler growth on carcass traits and meat quality in wild male red deer harvested by selective stalking
Source: PLoS One. 2026 Jul 8;21(7):e0352456. doi: 10.1371/journal.pone.0352456 (PMC13345254; doi:10.1371/journal.pone.0352456)
Supplement: Attachment — Submitted filename: GENERAL COMMENT.pdf [file pone.0352456.s002.pdf]

REVIEWER'S COMMENTS

I. GENERAL COMMENT

The work is solid, with original data and a generally good structure; however, the manuscript lacks a Conclusion section, and the order of sections does not follow standard conventions. Please reorganize it as follows: Introduction, Methods, Results, Discussion, and Conclusion. In addition, make sure to clearly name and number each section and subsection (for example: 1. Introduction; 2. Materials and Methods; 2.1. Study Area; 2.2. Data Collection, etc.). For detailed formatting requirements, please refer to the PLOS ONE submission guidelines.

https://journals.plos.org/plosone/s/submission-guidelines?

1. Lines 1–3

Overlong; “killed stress-free” is emotive; I suggestion you to change the title to this

Effects of season and antler growth on carcass traits and meat quality in wild male red deer harvested by selective stalking

2. Line 4-23: Put it in English

3. Abstract (lines 24–39)

According to the PLOS submission guidelines, the abstract must not exceed 300 words.

https://journals.plos.org/plosone/s/submission-guidelines?

a) Line 25–27: “This study examines for first time…” put this one “This study examines, for the first time, the effects…”

b) Line 28–29: “…on loin muscle quality from 32 adult wild male deer culled stress-free.”

c) Line 30–33: “summer; $p < 0.001$) … ($p = 0.016$).” Ensure uniform style: “($p < 0.001$) … ($p = 0.016$).”

d) Line 34: “pH at 72 h post-mortem (pH72)”

e) Line 35–38: “composition seems to be caused by the mineral mobilization…” “mineral composition patterns were consistent with seasonal skeletal mobilization during antler growth: Ca and Mg were higher in April; Fe and Zn were lower in April and June.”

f) Line 38–39: “Thus, we can conclude that both, the season and the effort to grow antlers, affected…” “Both season and antler-growth effort affected meat quality and composition in wild male red deer.”

4. Keywords (line 41–42)

“Fatty acids; meat composition; quality traits; red deer; seasonality.”

5. Introduction (lines 44–88)

a) line 48–54: “In fact, currently, red deer is the main wild game meat…” “deer is” → “deer are”; redundant “in fact, currently”; “carbon fingerprint” “Red deer are a major game meat worldwide, and consumption has increased over the last decade [1,2]. Venison from wild or estate culls has a lower environmental impact and carbon footprint than meat from industrial livestock systems [3].”
b) line 55–60: “Season has an impact… hunting period influences growth [5], [6] …” Clustered numeric citations; use ranges when appropriate; tighten | “Season affects cervid meat quality, particularly in seasonal breeders [4–10]. Hunting period also influences growth and carcass traits [5–7].”
c) line 61–66: “In fact, antlers are the fastest growing tissue and constituting…” Grammar; “skeleton mass” “Antlers are the fastest-growing mammalian appendage and can represent up to 28% of skeletal mass, driving cyclical physiological osteoporosis in spring to mineralize antlers [11]. This may alter meat mineral composition [12].”
d) line 68–71 “the possibility that the anticancer properties…” Off-scope speculation in Intro Delete from Introduction or move as a brief, clearly speculative note to Discussion.
e) line 72–79: “Monteria… thus producing a stressful death), or non-stressful death…” | Emotive language; define neutrally | “Common methods in Spain include Montería (driven hunts with dogs) and stalking (selective culling without chase). Driven hunts can increase pre-mortem stress relative to stalking [12].”
f) line 82–88: Aim paragraph Redundant season labels “We tested the effect of hunting month (September, January, April, June) on carcass and meat traits of wild red deer males culled by stalking, avoiding confounding by hunting stress.”

6. RESULT

- Add at least one figure showing **seasonal carcass weight/yield** with mean ± 95% CI.
- Add one figure showing **key mineral** or **FA indices** by month.

6.1. Table 1 — BW, carcass, pH, color, composition, cooking, texture

a) Recalculate **Chroma (C*)** for January; it’s inconsistent with the a* and b* you reported. correct January value (~19). Add footnote: “C* computed from mean a*, b*.”

b) **Hue (H)**: your values are labeled in degrees but look like radians. Either convert to degrees or relabel the unit. Then recheck the p-value.

c) Align your **text** with the table: don’t claim “no color effect” if **Hue** is significant. Specify exactly which color variables are/aren’t affected.

d) **Shear force**: fix the **unit**. Report Warner–Bratzler in **Newtons** (or kgf) consistently, not “kg/cm^2.” If any values were in a different unit, convert and rerun the stats and post-hoc letters.

e) Provide **mean ± SD** (not just SEM) and name the **post-hoc test** (e.g., Tukey HSD). PLOS prefers mean ± SD for interpretability

f) f) Line 99, Table 1 must be mentioned in the interpretation.

6.2. Table 2 Minerals (mg/100 g muscle)

a) Don't say the month affected **all** minerals. **Cu** is not significant; say "most minerals."

b) Investigate the **April Ca** spike. Verify lab notes, dilutions, and potential **bone contamination**. If confirmed as an outlier, justify it with QC and a sensitivity analysis, or correct the value if it was a transcription error.

c) Explain the large month-to-month variability in **Na** (management details like supplemental feed/salt access or transport conditions). Add a brief limitations note if you cannot document it.

d) Report **mean ± SD**, LOD/LOQ, and any **CRM** or recovery data used for ICP-OES.

e) Line 115-116: "Potassium (K) was the most abundant micromineral (42.8% of total), followed by P (35.6%) and Na (13.7%)."

6.3. Table 3: Fatty acids in mg/100 g muscle

a) Do not imply a significant difference for **DHA** on this basis if the p-value is not significant.

b) Standardize **isomer labels** (e.g., C18:1 t11, not "C 18:1-11t"). Remove stray spaces and unify notation.

c) Acknowledge and explain the **mismatch** between total FA per 100 g muscle and **IMF** from Table 1 (different subsamples, FAME→FA conversion, within-muscle variability). Make this explicit in Methods/Discussion.

6.4. Table 4: Total FA (mg/100 g) and aggregate indices

a) Fix number formatting (thousands separator) and consistent "**p-value**" styling.

b) In the text, remind readers this table is on a **muscle basis (mg/100 g)**, which can yield different significance patterns from **percentage basis** in Table 5.

6.5. Table 5: Fatty acids as % of total FA (g/100 g FA)

a) Correct the impossible **C18:0** percentage in **January**. A component cannot exceed the class total. Recompute from your mg/100 g data.

b) Verify unusually high **C15:1 n-5** values. Check identification against standards, consider coelution, and confirm with additional QC (and revise if misassigned).

c) Clean up **nomenclature** for trans isomers and spacing.

d) After any correction, **recalculate totals, ANOVA, and post-hoc letters** as needed.

6.6. Table 6: Indices (percentage basis)

a) These indices depend on Table 5. After you fix Table 5, **recompute** all indices and confirm significance and letters.

b) Add the **explicit formulas** for h/H, IA, IT in a footnote with the proper references. Keep notation consistent.

6.7. Table 6 interpretation must do (and currently doesn't)

1. **State the data basis up front.**
   Say clearly that Table 6 indices are calculated on a **percentage basis** (g/100 g of total FA, i.e., depend on Table 5), not on mg/100 g muscle. Your text mixes bases elsewhere; don't do that here.

2. **Name the winners/losers for each index, with direction and meaning.**
   For each line in Table 6, you must explicitly say which month is highest/lowest and what that implies:

   - **PUFA/SFA:** January highest; others much lower. Spell out that this indicates a **more favorable lipid profile** in January.
   - **Σn-6:** January highest.
   - **Σn-3:** January highest.
   - **n-6/n-3:** January highest; April lowest. Explain the logic: ratio rises in January because n-6 increases more than n-3.
   - **Long-chain n-3 (EPA+DPA+DHA):** January highest; April/June lowest. Note the **nutritional relevance**.
   - **Nutritional value (NV = Σ[C12:0+C14:0+C16:0]/Σ[C18:1n-9+C18:2n-6]):** April and June highest, January lowest. Add the interpretation: **higher NV = less favorable**.
   - **h/H:** January maximal. Say explicitly: **higher h/H = better**.
   - **IA:** January minimal; April/June maximal. Say: **lower IA = better**.
   - **IT:** January minimal; others higher. Say: **lower IT = better**.

3. **Stop referencing Table 6 for percentages of individual FA.**
   Your current paragraph drifts into single-FA percentages and even cites Table 6 for that.

Table 6 is **indices only**. Keep individual FA percentages in **Table 5** and quantities in **Table 3/4**.

4. **Align claims with p-values.**
   If you claim an index "differs among months," you must reference the **Table 6 p-value** for that index. Do not imply significance where it isn't shown.

5. **Add the formulas and references.**
   In a footnote (or Methods), include the explicit definitions with citations (Santos-Silva for **h/H**; Ulbricht & Southgate for **IA** and **IT**). If you don't show formulas, reviewers will assume hand-waving.

6. **Acknowledge dependency on Table 5 corrections.**
   Your indices inherit errors from Table 5. You must say you will **recompute** the indices and recheck significance and letter codes after fixing Table 5 (e.g., the impossible January **C18:0** percentage; the suspiciously high **C15:1 n-5** identification).

7. Discussion (lines 189–414)

| Lines | Original | Issue | Correction |

|---|---|---|

| 189–196 | Long consumer intro | Wordy; keep short | Compress to two sentences focusing on supply, seasonality barrier, farmed venison comparison. |

| 213–214 | "we designed current study…" | Article; clarity | "we designed the present study to isolate season effects by culling animals by stalking in all months." |

| 234–252 | June selection bias argument | Speculative; support with age data already given | Tie to ages (line 459): "September males were older (8.4 ± 2.8 y) than June (2.8 ± 0.5 y), consistent with selection against high-trophy prospects in June; this may partly explain lower BW/carcass yield." |

| 272–299 | pH interpretation | Fine; trim repetition; emphasize no DFD | Keep core; remove duplicated literature exposition |

| 332–364 | Minerals mechanism | Good idea; but April Ca outlier must be validated | Add limitations: lack of concurrent serum/bone biomarkers; muscle is an indirect proxy |

| 366–413 | FA context | Tighten; ensure WHO/FAO claim is referenced correctly; remove double period at [36].. | Fix punctuation; ensure every claim has a citation |

Add a short "Limitations" paragraph: single estate; supplementation 300–500 g day^{-1} is a confounder; age structure differs by month; no diet composition quantified; no serum/bone markers.

8. Methods (lines 415–end)

Instead of Study Plan, you should use Study Area. You need to clearly describe the study area so that readers can easily understand it. Include a map showing the location of your study area. Describe the area in detail, situating the park in relation to the nearby city and the country (Spain), and provide relevant climatic information.

After that, you can add a section titled Data Collection, where you will explain how the data were gathered.

| Lines | Original | Issue | Correction |

418–420: "38º53'N, 4º17'E" | West longitudes in Spain; likely W | "38°53′N, 4°17′W" |

421–426: Plant taxa: "Pistacea Lentiscus…" | Genus/species capitalization and italics | Pistacia lentiscus, Pistacia terebinthus, Cistus ladanifer, Phillyrea angustifolia, Salvia rosmarinus (formerly Rosmarinus officinalis), Quercus ilex, Erica spp., Genista spp. |

425–426:"Supplementary food… 300–500 g per deer and day." | Major confounder; must state composition and brand | Add feed composition (protein, fat, mineral profile), manufacturer, and confirm access was equal across months |

431–433: | Weather means | Fine; note data source | Add data source (station ID) |

436–437 : "stalking (sudden-low stress death that did not involve a chase by dogs)" | Hyphenation; neutrally describe | "stalking (selective culling without chase)" |

452–460 :Age determination; decimals with commas | Use decimal points; report agreement | Replace 2,9 with 2.9, etc. Add inter-rater agreement (e.g., 84% exact; Cohen's κ if available) |

462–479:Sampling, pH meter brand | Good; include probe model, calibration details already there | Ensure consistent manufacturer locations; keep T and pH calibration details |

Colour methods :"Objective measures of meat colour [50]…" | Specify device/illuminant/observer/bloom | Add: spectrophotometer model, D65, 10° observer, aperture, 1 h bloom, 3 readings averaged |

| Statistics | Not present in provided lines | PLOS requires full plan | Add: Shapiro–Wilk for normality; Levene for homoscedasticity; one-way ANOVA (factor: month) with Tukey HSD; $\alpha = 0.05$; report η^2 and 95% CIs; software/version (R 4.x with packages or SPSS v26) |
