# Supplementary material for: Effects of season and antler growth on carcass traits and meat quality in wild male red deer harvested by selective stalking
Source: PLoS One. 2026 Jul 8;21(7):e0352456. doi: 10.1371/journal.pone.0352456 (PMC13345254; doi:10.1371/journal.pone.0352456)
Supplement: Attachment — Submitted filename: Response to reviewers PONE-D-25-30036.docx [file pone.0352456.s003.docx]

Reviewer Report: PONE-D-25-30036

Manuscript Title: [Effect of hunting month and physiological effort of antler growth

on carcass and meat quality of wild red deer males killed stress-free]

Journal: [PLOS ONE]

**Reviewer 1 Report**

Summary of the Research and Overall Impression

The manuscript explores the influence of hunting month and the physiological demands of antler growth on carcass and meat quality traits in wild red deer. The topic is original and highly relevant to wildlife physiology, game meat science, and ecological management. The study addresses a novel research question that integrates seasonality with metabolic and mineral dynamics in deer. Overall, the work is promising and potentially valuable, but several sections require clearer structuring, refinement of interpretation, and improved scientific precision to meet PLOS ONE standards.

**Major Comments**

**Authors:** We appreciate the reviewer's suggestions and will detail the changes made one by one.

**Reviewer 1 comment:** (Lines 27–29) The sentence “This study examines for first time the effects of seasonality…” should be corrected for grammar (e.g., “for the first time”) and revised for clarity. The authors should also state their specific hypotheses or objectives explicitly to strengthen the abstract’s focus.

**Authors:** Corrected. “This study aimed to evaluate, for the first time, how seasonality—assessed by hunting month …”

**Reviewer 1 comment:** (Lines 25–39) The abstract currently blends background, results, and interpretation. A more structured flow would improve readability:

1–2 sentences for background/context;

1 sentence for the main aim;

2–3 sentences summarizing key findings with direction and significance;

1 concluding sentence emphasizing novelty or implications.

**Authors:** We have modified the structure and clarified the context of the entire abstract to address the reviewer's suggestion.

**Reviewer 1 comment:** (Lines 31–34) While including p-values is good practice, ensure consistency (use “p < 0.05” uniformly). Mentioning the general type of statistical test (e.g., ANOVA or mixed model) would help readers appreciate the analytical rigor.

**Authors:** We have modified the p-values in the abstract and standardised them to p < 0.05.

**Reviewer 1 comment:** Interpretation vs. Data Presentation (Lines 35–38) Avoid causal phrasing such as “caused by.” Since this is observational research, use softer wording like “may be influenced by” or “is likely associated with” to align with descriptive study conventions.

**Authors:** We have modified the sentence: “…mineral composition may be more strongly associated with skeletal mobilization during antler growth than with dietary variation: calcium and magnesium concentrations were elevated in April, while iron and zinc levels were reduced in April and June.”

**Minor Comments**

**Reviewer 1 comment:** • Maintain consistent spacing around parentheses and symbols (e.g., p < 0.05).

**Authors:** Corrected

**Reviewer 1 comment:** • Replace “culled stress-free” with “harvested under minimal stress conditions” for more formal scientific tone.

**Authors:** Corrected

**Reviewer 1 comment:** • Clarify “loin muscle quality” by identifying the specific muscle studied (e.g., Longissimus thoracis et lumborum).

**Authors:** Corrected

**Reviewer 1 comment:** • Strengthen the concluding line by emphasizing novelty, e.g., “These findings demonstrate that seasonal dynamics and physiological investment in antler growth jointly influence meat composition in wild red deer, offering new insights into nutritional ecology and game meat quality.”

**Authors:** Included

**Introduction**

**Reviewer 1 comment:** (Lines 45–71) The introduction combines several themes—environmental sustainability, deer biology, antler physiology, and hunting practices—without a clear logical progression. Consider restructuring:

• Paragraph 1: Importance of venison as a sustainable protein source.

• Paragraph 2: Seasonal physiological effects on meat quality.

• Paragraph 3: Mineral mobilization and antler growth.

• Paragraph 4: Hunting context and stress effects.

• Final paragraph: Explicitly state study aims and hypotheses.

**Authors:** Corrected

**Reviewer 1 comment:** (Lines 82–88) The novelty and objectives should be emphasized more clearly to justify the study’s rationale.

**Authors:** Corrected.

**Reviewer 1 comment:** (Lines 61–67) The discussion of “cyclic osteoporosis” is interesting but overly detailed for the Introduction. Condense this section and refer readers to the cited review ([11]) for further context.

**Authors:** Corrected

**Reviewer 1 comment:** (Lines 68–71) The statement that antler-derived biomolecules may have anti-aging or anticancer properties is speculative. It should be softened (e.g., “It has been suggested that…”) or omitted to maintain scientific objectivity.

**Authors:** Corrected

Minor Notes

**Reviewer 1 comment:** • Define abbreviations such as FA (fatty acids) upon first mention. **Authors:** Corrected

We have included “*Table 4 (new numbering after including table 1 for feed composition). Effect of hunting month on saturated (SFA), monounsaturated (MUFA), and polyunsaturated (PUFA) fatty acid (FA) profile (mg/100 g of muscle) of meat from adult red deer males*”

**Reviewer 1 comment:** • Use the species name “red deer (Cervus elaphus)” consistently.

**Authors:** Corrected

**Reviewer 1 comment:** • Check reference formatting (use either “(1,2)” or “[1,2]” per journal style).

**Authors:** Corrected

**Reviewer 1 comment:** • Ensure that all numerical or quantitative claims are properly cited.

**Authors:** Revised

**Results**

**Reviewer 1 comment:** (Lines 132–180) The observed seasonal variations are clearly presented, but biological interpretation should be strengthened. The authors should explicitly link observed trends (e.g., higher carcass yield in September) to nutritional status and antler growth demands, rather than presenting them descriptively.

**Authors:** The section and text lines the referee indicates refers to results. Results have to be descriptive. It is in discussion where we can link results to nutritional status, antler mineral mobilization per season, etc. to interpret the results. PLoS ONE is not a journal that mixes results and discussion in one section.

**Reviewer 1 comment:** (Lines 181–210) Mineral variation results (Ca, Mg, Fe, Zn) are intriguing but require deeper explanation. Discuss how these results relate to bone mobilization and antlerogenesis, citing relevant literature on mineral metabolism in cervids.

**Authors:** As in the previous comment, the results section only presents the results, and the interpretation is rather deeply addressed in the discussion (old version: lines 332-364; revised version: lines 524-556).

**Reviewer 1 comment:** (Lines 211–250) The section on fatty acid profiles can be condensed. Focus on the biological implications of PUFA/SFA ratios and related health indices, rather than excessive numerical details.

**Authors:** As with mineral content, the biological implications, as well as those regarding meat quality (which is part of the audience of the article) are addressed in the discussion in lines 558-605 of the new version. The results show the actual composition. It may look lengthy for a reader that is not interested in fatty acids, but we also have to think that meat scientists/nutritionist may be interested in a particular fatty acid, and the information has to be complete (particularly considering that now, electronic pdfs do not imply a cost of paper printed, and lengthy data can just be skipped by some readers, unlike in printed journals in the past).

**Reviewer 1 comment:** • Ensure uniform significance notation (p < 0.05).

**Authors:** we found that some p were italics and others were not. We have unified all instances now with the same style.

**Reviewer 1 comment:** Clarify if post-hoc corrections were applied for multiple testing.

**Authors:** Sorry, the referee is right: we forgot to mention this, but we did a Tukey test. The sentence in lines 278-280 is now “To discriminate which groups differ from others, as shown on tables, a Tukey test was performed.”

**Reviewer 1 comment:** Minor Suggestions Group related fatty acid indices or mineral results into summary tables to improve clarity and readability.

Discussion

**Reviewer 1 comment:** (Lines 189–410) The Discussion is lengthy and partially repetitive. Consider condensing it by removing background information already presented in the Introduction.

**Authors:** We have reduced it in several parts (in the fatty acid section).

**Reviewer 1 comment:** Each results subsection (carcass, meat traits, minerals, FA profile) is discussed in isolation; an integrative paragraph linking these findings through nutrition–antler growth– metabolism interactions would enhance coherence. Some statements (e.g., hunter bias, lines 244–250) are speculative and should be rephrased cautiously unless supported by data. Also, the Discussion relies heavily on the group’s own previous studies [10, 12]. A broader comparison with international studies would improve generalizability.

**Authors:** Starting from the end, we compare our results with those international ones in some aspects, but the point is that our own studies were conducted on the same game estate, with the same laboratory equipment and with many confounding variables being equal. Wherever we can, we compare our results with others. An example is the meat characteristics (lines 510-515): “…the shear force observed in the current study was (…) lower than (…) Cawthorn et al. [26] (…) similar to (…) observed by Piaskowska et al. [27] (…) and (…) by Maggiolino et al. [28]”. However, but the effects we are discussed in our article are directly related to previous study in the same game estate with season and type of hunting/stress combined producing a confounding effect. Regarding reference 11 and others by our group, this is the most comprehensive review on antlers, and our group is the leading group in the world in hard antler science (see ranking of TLC as 5^th^ in the world rank here: <https://expertscape.com/ex/antlers>; the first 4 work in stem cell biology of the growing antler), so that it is not surprising we cite our papers.

Regarding “an integrative paragraph linking these findings through nutrition–antler growth– metabolism interactions would enhance coherence” we use this approach wherever possible, but we cannot fully do this, for example, in fatty acid composition. In addition, there are other approaches that some readers in meat science, for example, may want to see: “how does fatty acid composition relate to that published in meat science studies on NZ farmed deer”, for example.

Regarding statements on hunter bias, we know it is speculative and we actually point it like this, as a possibility, not a proven-fact.

Minor Adjustments

**Reviewer 1 comment:** • Simplify overly long sentences (e.g., lines 300–310).

**Authors:** Those sentences are normal. The only ones that are long, but still reasonable are those in 510-515: “*As an average, the shear force observed in the current study was 22.2 N, a value much lower than the 30.2 N observed by Cawthorn et al. [26], for the LTL muscle from male wild fallow deer. However, these values were similar to the 22.1 (stags) and 19.2 (hinds) N observed by Piaskowska et al. [27] for the Longissimus lumborum muscle from wild fallow deer and to the 19.2 N observed by Maggiolino et al. [28] as average for the LTL muscle from male wild red deer with different ages at slaughter.*” However, if the referee can point exactly a very long sentence or group of sentences, we are ready to shorten them.

**Reviewer 1 comment:** • Standardize terminology (IMF, intramuscular fat, slaughter BW). **Authors:** Corrected

**Reviewer 1 comment:** • End with a concise, practical statement highlighting management implications, e.g., “Identifying the optimal hunting period could enhance both meat quality and resource sustainability.”

• Materials and Methods

**Authors:** Good suggestion. It is not so easy, as the hunting period is basically autumn-winter, adding a small proportion for population reduction, but we have added this sentence: “*Understanding the seasonal effects in meat quality and antler cycle mobilization of minerals may help to take decisions to balance hunting party or population density reduction with meat quality variations for its marketing*.”

**Reviewer 1 comment:** (Lines 427–460) Although the design is balanced (n = 8 per month), age varies notably among months (~8 years in September vs. ~3 years in others). This age difference is a major confounder that could influence carcass and meat traits. Clarify whether age was statistically controlled (e.g., as a covariate), and if not, acknowledge this limitation explicitly.

**Authors:** The numbers per month are low. We are already short in sample size (but each animal is worth 1200 € or more, so we cannot ask for a larger sample, and adding a covariate requires more observations for the analysis to be acceptable. With these strong constraints, we have tried to explain in the new version adding the following sentence (lines 155-157): “*Unfortunately, shooting depends on the opportunity of an animal appearing on view of the hunter, age cannot be easily determined by sight, and thus, it cannot be controlled easily as in a farm*”.

**Reviewer 1 comment:** (Lines 572–579) The General Linear Model includes only “hunting month” as a fixed factor. Given the complexity of biological variation, age and possibly environmental factors (temperature, rainfall) should be considered covariates. Indicate whether post-hoc tests were adjusted for multiple comparisons (e.g., Tukey HSD).

**Authors:** As explained above, the more variables we add, the more observations we need, therefore we made as appropriate to the sample size as possible. The GLM is a regression not based on multiple comparisons as a set of ANOVAs. However, the reviewer is right in that we used multiple comparisons in tables to show which groups are different from each other, and these are not explained in the methods. We have added this sentence now (lines 278-280): “*To discriminate which groups differ from others, as shown on tables, a Tukey test was performed.*”

**Reviewer 1 comment:** (Lines 463–466). Specify the time interval between death and muscle sampling, as this directly affects pH and biochemical parameters. Clarify whether carcasses were cooled before sampling or analyzed fresh.

**Authors:** We have clarified this now in the following sentence 155-157: “*This means that the time elapsed between death and laboratory analysis was less than 24h, and most of the time the samples were stored at about 5ºC*”

Minor Comments

**Reviewer 1 comment:** • Use decimal points consistently (e.g., 2.9 ± 0.7 years).

**Authors:** Corrected

**Reviewer 1 comment:** • Correct typographical error: “cocked meat weight” → “cooked meat weight.”

**Authors:** Corrected

**Reviewer 1 comment:** • Ensure full citation details for AOAC and ISO references (e.g., publication year, method number).

**Authors:** Probably the referee means AOCS and ISO. However, in the references are the details he mentions, and these references were already in the MS. If there is something we did not understand, please clarify and we will change it.

56.	AOCS. Official Procedure Am 5-04. Rapid determination of oil/fat utilizing high temperature solvent extraction. American Oil Chemist´s Society Urbana, IL, USA. 2005.

53.	ISO 937. International standards meat and meat products. Determination of nitrogen content. International Organization for Standardization. Geneva, Switzerland. 1978.

54.	ISO 1442. International standards meat and meat products. Determination of moisture content. International Organization for Standardization. Geneva, Switzerland. 1997.

55.	ISO 936. International standards meat and meat products. Determination of ash content. International Organization for Standardization. Geneva, Switzerland. 1998.

# **REVIEWER 2 COMMENTS**

**I. GENERAL COMMENT**

**Reviewer 2 comment:** The work is solid, with original data and a generally good structure; however, the manuscript lacks a Conclusion section, and the order of sections does not follow standard conventions. Please reorganize it as follows: Introduction, Methods, Results, Discussion, and Conclusion. In addition, make sure to clearly name and number each section and subsection (for example: 1. Introduction; 2. Materials and Methods; 2.1. Study Area; 2.2. Data Collection, etc.). For detailed formatting requirements, please refer to the PLOS ONE submission guidelines.

[https://journals.plos.org/plosone/s/submission-guidelines?](https://journals.plos.org/plosone/s/submission-guidelines)

**Authors:** corrected.

**Reviewer 2 comment:** Lines 1–3. Overlong; “killed stress-free” is emotive; I suggestion you to change the title to this “*Effects of season and antler growth on carcass traits and meat quality in wild male red deer harvested by selective stalking*”.

**Authors:** Stress-free is not emotive, it is a scientific description, as we say in another part of this letter. Stress is part of animal life. An animal chased by dogs (or wolves) in a situation that can die has stress and the body reacts by stress-hormones (adrenaline, cortisol) to focus whole phisiology and muscle reaction in scaping to survive. An animal killed suddenly by a shot in the head probably does not even hear it because the brain destruction is faster than the sound. The body does not have time to react in stress. In any case we have changed the title.

**Reviewer 2 comment:** Line 4-23: Put it in English.

**Authors:** corrected as suggested.

**Reviewer 2 comment:** Abstract (lines 24–39). According to the PLOS submission guidelines, the abstract must not exceed 300 words. [https://journals.plos.org/plosone/s/submission-guidelines?](https://journals.plos.org/plosone/s/submission-guidelines)

**Authors:** corrected as suggested. It has now 255 words.

**Reviewer 2 comment:**

a) Line 25–27: “This study examines for first time…” put this one “This study examines, for the first time, the effects…”.

**Authors:** Not applicable now, as shortening has changed the sentence to “This study evaluated how seasonality…”

**Reviewer 2 comment:**

b) Line 28–29: “…on loin muscle quality from 32 adult wild male deer culled stress-free.”

**Authors:** to accommodate the suggestions of another referee we changed the sentence to “the quality of the Longissimus thoracis et lumborum muscle in adult wild male red deer harvested under minimal stress conditions”.

**Reviewer 2 comment:**

a)	Line 30–33: “summer; p < 0.001) … (p = 0.016).” Ensure uniform style: “(p < 0.001) … (p = 0.016).”

**Authors:** we have revised all p throughout the MS to make a uniform style.

**Reviewer 2 comment:**

c) Line 34: “pH at 72 h post-mortem (pH72)”.

**Authors:** Corrected as proposed.

**Reviewer 2 comment:**

d) Line 35–38: “composition seems to be caused by the mineral mobilization…” “mineral composition patterns were consistent with seasonal skeletal mobilization during antler growth: Ca and Mg were higher in April; Fe and Zn were lower in April and June.”

composition seems to be caused by the mineral mobilization from the skeleton to grow antlers. , as Ca and Mg were higher in April, and Fe and Zn were lower in this month and June

**Authors:** With the reduction of the abstract the sentence changed slightly, but we kept the part that the referee points: “*Mineral composition exhibited a distinct pattern, suggesting a stronger association with skeletal mobilization during antler growth than with dietary changes. Mineral composition patterns were consistent with seasonal skeletal mobilization during antler growth: Ca and Mg were higher in April; Fe and Zn were lower in April and June*.”

**Reviewer 2 comment:**

e) Line 38–39: “Thus, we can conclude that both, the season and the effort to grow antlers, affected…” “Both season and antler-growth effort affected meat quality and composition in wild male red deer.”

**Authors:** Again, slightly changed but including the remarks by the referee: “*Overall, these findings demonstrate that both season and antler-growth effort affected meat quality and composition in wild male red deer, providing new insights into nutritional ecology and game meat quality*.”

**Reviewer 2 comment:**

Keywords (line 41–42). “Fatty acids; meat composition; quality traits; red deer; seasonality.”

**Authors:** corrected for capitals.

**Reviewer 2 comment:**

Introduction (lines 44–88)

a) line 48–54: “In fact, currently, red deer is the main wild game meat…” “deer is” → “deer are”; redundant “in fact, currently”; “carbon fingerprint” “Red deer are a major game meat worldwide, and consumption has increased over the last decade [1,2]. Venison from wild or estate culls has a lower environmental impact and carbon footprint than meat from industrial livestock systems [3].”

**Authors:** All changes possible included, although as a result of the comments by other reviewers, the text changed slightly. We also improved the phrasing. The text is now: “*Red deer (Cervus elaphus) are a major large game species hunted in Europe, as they are widely distributed and highly abundant. Thus, they constitute probably the main game meat worldwide, and consumption has increased over the last decade [1, 2], particularly in touristic cities in central and northern Europe. Venison from wild or estate culls has a lower environmental impact and carbon fingerprint than meat from industrial livestock systems, helping to maintain biodiversity conservation and rural development [3].*”

**Reviewer 2 comment:**

b) line 55–60: “Season has an impact… hunting period influences growth [5], [6] …” Clustered numeric citations; use ranges when appropriate; tighten | “Season affects cervid meat quality, particularly in seasonal breeders [4–10]. Hunting period also influences growth and carcass traits [5–7].”

**Authors:** Changed approximately as suggested. New text is now “Season affects cervid meat quality, particularly in seasonal breeders [4–10]. (…) Deer hunting period also influences growth [5, 6], carcass traits [7], and meat quality [7-10].”

**Reviewer 2 comment:**

c) line 61–66: “In fact, antlers are the fastest growing tissue and constituting…” Grammar; “skeleton mass” “Antlers are the fastest-growing mammalian appendage and can represent up to 28% of skeletal mass, driving cyclical physiological osteoporosis in spring to mineralize antlers [11]. This may alter meat mineral composition [12].”

**Authors:** We have addressed the improvements where we got it wrong (skeletal mass instead of skeleton). We thank the reviewer in the effort for such improvements. However, being experts in antlers, the rest of the text is how we want to express it with the level of detail we think is required. The new text is “*Antlers are the fastest-growing mammalian tissue and can represent up to 28% of skeletal mass. Antler mineralization induces a process known as cyclical physiological osteoporosis, where minerals are mobilized from the skeleton to support antler development (reviewed in Landete-Castillejos et al. [11]). This process may influence the mineral profile of meat, particularly in spring-hunted animals [10, 12-13]*.”

**Reviewer 2 comment:**

d) line 68–71 “the possibility that the anticancer properties…” Off-scope speculation in Intro Delete from Introduction or move as a brief, clearly speculative note to Discussion.

**Authors:** Deleted as suggested.

**Reviewer 2 comment:**

e) line 72–79: “Monteria… thus producing a stressful death), or non-stressful death…” | Emotive language; define neutrally | “Common methods in Spain include Montería (driven hunts with dogs) and stalking (selective culling without chase). Driven hunts can increase pre-mortem stress relative to stalking [12].”

**Authors:** Thanks for the suggestion, but it is not emotive. Stress exists in animals, producing a high level of cortisol. It is not emotive to think that animals chased by dogs that often bite and kill an animal (remember that a dog is Canis lupus familiaris, a subspecies from the wolf!) suffer stress. In the meat science it is well established the effects on the meat quality of a stressful/non stressful death. Sudden death by a shot in the head does not produce stress. The physical effort of running and the sudden hormonal changes induced by cortisol and adrenaline have consequences for chemical and physical qualities of the meat. Deleting mention to stress would weaken the implications of the study.

**Reviewer 2 comment:**

f) line 82–88: Aim paragraph Redundant season labels “We tested the effect of hunting month (September, January, April, June) on carcass and meat traits of wild red deer males culled by stalking, avoiding confounding by hunting stress.”

**Authors:** We have discarded redundant terms during an overall change in the text. This part is now: “*Therefore, the aim of this study was to evaluate how seasonality—assessed by hunting month—and the physiological effort of antler growth affect carcass traits and meat composition in adult wild male red deer harvested under minimal stress conditions. We hypothesized that seasonal changes in food availability and mineral mobilization during antlerogenesis would be associated with significant variation in meat quality parameters.*”

**Reviewer 2 comment: RESULTS.**

**-Add at least one figure showing seasonal carcass weight/yield with mean ± 95% CI.**

**-Add one figure showing key mineral or FA indices by month.**

**Authors:** We thank the referee for this suggestion, but the article is already very long, and adding figures of data that are already on tables, is enlarging it without a real need. The information suggested and much more is already presented on tables, and readers can figure out what the trends would be. Doing a graphic for some minerals or FA and not others would not be justified for some readers (or us).

**Reviewer 2 comment:** Table 1 — BW, carcass, pH, color, composition, cooking, texture

a) Recalculate **Chroma (C*)** for January; it’s inconsistent with the a* and b* you reported. correct January value (~19). Add footnote: “C* computed from mean a*, b*.”

**Authors:** Sorry, we are not substituting a measurement by a means of two measurements in other months. We think is incorrect. All the measurements were conducted by the same trained people.

b) **Hue (H)**: your values are labeled in degrees but look like radians. Either convert to degrees or relabel the unit. Then recheck the p-value.

**Authors:** the referee is right. The value 0.60 would be nearly red if in degrees, whereas in radians matches the colour of the fresh meat. We have changed from Hº to “Hue angle (H rad)”.

c) Align your **text** with the table: don’t claim “no color effect” if **Hue** is significant. Specify exactly which color variables are/aren’t affected.

**Authors:** sentence changed to (note the new numbering) “*As shown on Table 2, hunting period did not affect lightness, redness, yellowness, or chroma, although it did affect Hue angle, indicating that the overall colour intensity did not change, but the colour nuance (i.e., the angle defined by a* and b*) shifted slightly.*”

d) **Shear force**: fix the **unit**. Report Warner–Bratzler in **Newtons** (or kgf) consistently, not “kg/cm².” If any values were in a different unit, convert and rerun the stats and post-hoc letters.

**Authors:** We have changed kg to N, and, as suggested, we re-run an ANOVA with t Tukey test. In addition, in this and the rest of the tables, we have renamed non-significant values P above 0.1 that were shown by its exact probability to a hyphen “-“, as it does not matter whether the level of non-significance is p = 0.286 or p = 0.437. Now is less messy.

e) Provide **mean ± SD** (not just SEM) and name the **post-hoc test** (e.g., Tukey HSD). PLOS prefers mean ± SD for interpretability.

**Authors:** We disagree with the referee. The two statistics of dispersion mean different things. SD is a measure of the dispersion of a population. For example, if we measure the height of all humans, we give a means ± SD. That is why SD is included in description of the animals used in methods (e.g. age of the animals used, their weight, etc.). However, the standard error of the mean, SEM or SE, is an estimator of the population mean when the mean of the whole population is estimated by a sample n, and shrinks to 0 when the sample is very large SEM = SD/√n. That is why, in research, the results have to be shown on SEM because what we intend is to estimate where the true value of the variable mean is.

f) f) Line 99, Table 1 must be mentioned in the interpretation.

**Authors:** changed as suggested “*Concerning physicochemical parameters (Table 2)*”. This is ((new numbering).

**Reviewer 2 comment:**

# 1.2. Table 2 Minerals (mg/100 g muscle)

a) Don’t say the month affected **all** minerals. **Cu** is not significant; say “most minerals.”

**Authors:** Sorry but we cannot locate this statement to change neither in abstract, results on mineral content or in the section of discussion regarding mineral composition.

b) Investigate the **April Ca** spike. Verify lab notes, dilutions, and potential **bone contamination**. If confirmed as an outlier, justify it with QC and a sensitivity analysis, or correct the value if it was a transcription error.

**Authors:** We do not suspect there is an error here. We would expect an error if this occurred in winter or autumn, but in spring the antler is growing at a greater speed than cancer (1-4 cm/d in length, more than 20 cm^2^/d of skin in the tip of the main beam), and the mineralization is so intense (maximum of middle to large mammals) than the diet cannot supply the vast amount of Ca and other minerals needed, so the deer mobilize minerals from the skeleton in support of antler growth and they suffer osteoporosis (Landete-Castillejos et al. 2019. <https://doi.org/10.1016/j.bone.2019.115046>). Therefore, this spike is exactly what we would predict (also in June, but it seems that most of the minerals are mobilized in spring). There is no outlier here.

c) Explain the large month-to-month variability in **Na** (management details like supplemental feed/salt access or transport conditions). Add a brief limitations note if you cannot document it.

**Authors:** Animals are in the wild with little supplementation per head. There is no big change in management across months.

d) Report **mean ± SD**, LOD/LOQ, and any **CRM** or recovery data used for ICP-OES.

**Authors:** Please refer to our comment above about the differences between SD (descriptive of the whole population, to be reported in methods) and SEM (estimator of the mean, to be reported in results).

e) Line 115-116: “Potassium (K) was the most abundant micromineral (42.8% of total), followed by P (35.6%) and Na (13.7%).”

**Authors:** Thanks for the suggestion. Very simple now. Changed as suggested.

**Reviewer 2 comment:**

# 1.3. Table 3: Fatty acids in mg/100 g muscle

a) Do not imply a significant difference for **DHA** on this basis if the p-value is not significant.

**Authors:** We have changed non-significant trend in C20:3n-6 indicating “-“ instead of the non-significant probability. For the case the referee means, DHA at p = 0.085, we prefer not to change. At a small sample of 8 animals per month, a reader may conclude that a marginal significance level like this (P < 0.1) may become significant if the sample per month was 10 or 12 males. In any case we are not saying it is significant, we are just leaving the exact probability for values between 0.05 and 0.1.

b) Standardize **isomer labels** (e.g., C18:1 t11, not “C 18:1-11t”). Remove stray spaces and unify notation.

**Authors:** Thanks. The referee has a very sharp eye. This was the only case in which we made the error of adding a space. Corrected as suggested.

c) Acknowledge and explain the **mismatch** between total FA per 100 g muscle and **IMF** from Table 1 (different subsamples, FAME→FA conversion, within-muscle variability). Make this explicit in Methods/Discussion.

**Authors:** On the tables it is stated when the sample is expressed as mg/100g of muscle, and in the case of IMF it is the usual term of indicating the amount of fat inside the muscle. Maybe we do not fully understand, but we think it is clear as it is now.

**Reviewer 2 comment:**

# 1.4. Table 4: Total FA (mg/100 g) and aggregate indices

a) Fix number formatting (thousands separator) and consistent “**p-value**” styling.

**Authors:** corrected.

b) In the text, remind readers this table is on a **muscle basis (mg/100 g)**, which can yield different significance patterns from **percentage basis** in Table 5.

**Authors:** The text refers to the tables where appropriate, and in them it is stated that values refer on a muscle basis. We think there is little room for misunderstanding.

**Reviewer 2 comment:**

# 1.5. Table 5: Fatty acids as % of total FA (g/100 g FA)

a) Correct the impossible **C18:0** percentage in **January**. A component cannot exceed the class total. Recompute from your mg/100 g data.

**Authors:** We made an error with the decimal point. Corrected.

b) Verify unusually high **C15:1 n-5** values. Check identification against standards, consider coelution, and confirm with additional QC (and revise if misassigned).

**Authors:** verified. It is not an outlier.

c) Clean up **nomenclature** for trans isomers and spacing.

**Authors:** checked.

d) After any correction, **recalculate totals, ANOVA, and post-hoc letters** as needed.

**Authors:** addressed as appropriate.

**Reviewer 2 comment:**

# 1.6. Table 6: Indices (percentage basis)

a) These indices depend on Table 5. After you fix Table 5, **recompute** all indices and confirm significance and letters.
b) Add the **explicit formulas** for h/H, IA, IT in a footnote with the proper references. Keep notation consistent.

**Authors:** The change on Table 5 did not require new statistical. Regarding the formulae, the reviewer probably did not notice that the footnotes he is asking were in the following page (but belong to that table). These footnotes, already indicated the values he is asking. Copied and pasted from original (old version) are these:

^5^ Hypocholesterolemic/hypercholesterolemic ratio = [Σ(C18:1*n*‒7+C18:1*n*‒9+C18:2*n*‒6+C18:3*n*‒3+C20:3*n*‒6+C20:4*n*‒6)/Σ(C14:0+C16:0)].

^6^ Index of atherogenicity = [C12:0+4*C14:0+C16:0]/[(∑MUFA)+(∑PUFA)], with MUFA being the monounsaturated fatty acid.

^7^ Index of thrombogenicity = [C14:0+C16:0+C18:0]/[(0.5*∑MUFA)+(0.5*∑*n*‒6)+(3*∑*n*‒3)+(∑*n*‒3)/∑*n*‒6)], with MUFA being the monounsaturated fatty acid.

**Reviewer 2 comment:**

# 1.7. Table 6 interpretation must do (and currently doesn’t)

1. **State the data basis up front.**

Say clearly that Table 6 indices are calculated on a **percentage basis** (g/100 g of total FA, i.e., depend on Table 5), not on mg/100 g muscle. Your text mixes bases elsewhere; don’t do that here.

**Authors:** Corrected as suggested, but we think readers are intelligent and can understand even if not extra-clarified. The new text is now “(g/100 g of fatty acid; i.e. on a percentage basis)”

# 2. Name the winners/losers for each index, with direction and meaning.

For each line in Table 6, you must explicitly say which month is highest/lowest and what that implies:

- **PUFA/SFA:** January highest; others much lower. Spell out that this indicates a

**more favourable lipid profile** in January.

- **Σn-6:** January highest.
- **Σn-3:** January highest.
- **n-6/n-3:** January highest; April lowest. Explain the logic: ratio rises in January because n-6 increases more than n-3.
- **Long-chain n-3 (EPA+DPA+DHA):** January highest; April/June lowest. Note the **nutritional relevance**.

**Authors:** We have added now that we placed in bold the highest value: “Highest values in bold”. In any case, this is something that a reader with standard intelligence can find easily.

- **Nutritional value (NV = Σ[C12:0+C14:0+C16:0]/Σ[C18:1n-9+C18:2n-6]):**

April and June highest, January lowest. Add the interpretation: **higher NV = less favourable**.

**Authors:** We have added in footnote 4 “Higher value, less favourable. Lowest value in January”. Again, an educated reader that is interested in this can find it easily.

- **h/H:** January maximal. Say explicitly: **higher h/H = better**.
- **IA:** January minimal; April/June maximal. Say: **lower IA = better**.
- **IT:** January minimal; others higher. Say: **lower IT = better**.

**Authors:** An educated reader will know this, but in any case the referee is right in that it is little work to tell non-expert readers what is better. We have added on footnote 5 “Higher h/H = more healthy.” On footnote 6: “Lower IA = more healthy”. On footnote 7 Lower IT = more healthy.

**Reviewer 2 comment:**

# 3. Stop referencing Table 6 for percentages of individual FA.

Your current paragraph drifts into single-FA percentages and even cites Table 6 for that.

Table 6 is **indices only**. Keep individual FA percentages in **Table 5** and quantities in Table 3/4.

**Authors:** We have explained better following the suggestion of the referee. He is right in that by mentioning percentage before indexes, it may mislead the reader, and we have stated more clearly than the percentages are from table 6 (new numbering, old table 5). Now appear the word indexes earlier. The text is now: “*Table 7. Effect of hunting month on indexes of fatty acid (FA) profile (g/100 g of fatty acid; i.e. on a percentage basis of table 6) in meat from adult red deer males. Highest values in bold*”.

4. **Align claims with p-values.**

If you claim an index “differs among months,” you must reference the **Table 6 p-value**

for that index. Do not imply significance where it isn’t shown.

**Authors:** We have clarified the footnote “Different superscript letters within a row denote significant differences (p ≤ 0.05) in a Tukey test.”. In results we have limited the “differences between months” to “some months” and clarified “as shown on each table” for the reader to refer to each of them, although it was implied before. The text is now on results “which showed some differences among hunting months as shown on each table”

# 5. Add the formulas and references.

In a footnote (or Methods), include the explicit definitions with citations (Santos-Silva for **h/H**; Ulbricht & Southgate for **IA** and **IT**). If you don’t show formulas, reviewers will assume hand-waving.

**Authors:** As we mentioned earlier, probably the reader did not notice that the formulae were already present in the tables, but the footnotes were on the following page because only up to footnote 4 fitted the page, with footnotes 5-7 on the following page.

The references were already on the reference list as 65 and 66, but we have added them in the foot note: 5 (…). Ratio h/H calculated according to Santos-Silva et al. [65]. 6. (…) IA and IT were calculated according to Ulbricht and Southgate [66].

# 6. Acknowledge dependency on Table 5 corrections.

Your indices inherit errors from Table 5. You must say you will **recompute** the indices and recheck significance and letter codes after fixing Table 5 (e.g., the impossible January **C18:0** percentage; the suspiciously high **C15:1 n-5** identification).

**Authors:** The error in C18:0 was a decimal point misplaced when typing on the table, not on the SPSS. It was 7.1, not 71. No further statistical re-testing is required.

# 2. **Discussion (lines 189–414)**

**Reviewer 2 comment:**

| Lines | Original | Issue | Correction |

|---|---|---|

| 189–196 | Long consumer intro | Wordy; keep short | Compress to two sentences focusing on supply, seasonality barrier, farmed venison comparison. |

**Authors:** Following the suggestion of the first referee, we have modified the introduction.

**Reviewer 2 comment:**

| 213–214 | “we designed current study…” | Article; clarity | “we designed the present study to isolate season effects by culling animals by stalking in all months.” |

**Authors:** We have changed according to the suggestions of the referee, but keeping “stress-free” as we think it is important. The sentence now in lines 407-408 is “*we designed the present study to isolate season effects by culling animals always stress-free by stalking in all months*”.

**Reviewer 2 comment:**

| 234–252 | June selection bias argument | Speculative; support with age data already given | Tie to ages (line 459): “September males were older (8.4 ± 2.8 y) than June (2.8 ± 0.5 y), consistent with selection against high-trophy prospects in June; this may partly explain lower BW/carcass yield.” |

**Authors:** We did not find exactly the sentence the referee mentions, but he has a point and we have added age in the discussion regarding the lower body weight in June. The complete argument is now this, in lines 437-445 (the sentence modified is the last one); “*It is more difficult to explain why in June, after the recovery of BW during spring and with greater food availability, the slaughter BW, carcass weight, and yield are the second lowest. The reason maybe that in April the antler is just growing, but in June it is rather well developed, and it shows what type of trophy will be when it is fully grown and cleaned of skin in late August. In this situation, a hunter in a game estate will avoid killing animals that show potentially big trophies, so that, inadvertently, they may have killed adults of poorer trophy and younger (in fact, September males were older 8.4 ± 2.8 y than those in June 2.8 ± 0.5 y)*”

**Reviewer 2 comment:**

| 272–299 | pH interpretation | Fine; trim repetition; emphasize no DFD | Keep core; remove duplicated literature exposition |

**Authors:** Corrected as suggested. We moved DFD at the beginning and trimmed the text more than 50%: “*Seasonal changes have been reported to have effects on meat quality of deer [9], [11]. The final pH of deer meat analysed in the current study indicates a high quality of the product. In fact, no cases were detected for DFD percentage meat (final pH values above 6). There were, however, seasonal differences in pH: autumn and winter carcasses had values of pH (pH72) higher than those in spring and summer carcasses. Pérez-Serrano et al. [10], also did not find differences for pH between animals hunted in winter and autumn. As in the current study, Serrano et al. [12], found pH values in winter higher than those in summer, as Wiklund et al. [7] found in other conditions. Stress affects pH and thus, values in the present study by stalking were similar to stalking summer values but lower than stressful-chase in winter by Serrano et al. [12]*.”

**Reviewer 2 comment:**

| 332–364 | Minerals mechanism | Good idea; but April Ca outlier must be validated | Add limitations: lack of concurrent serum/bone biomarkers; muscle is an indirect proxy |

**Authors:** As mentioned above, we expect the peak in Ca in April because of skeleton mobilization in support of antler growth (maybe also in June, but we didn’t get it, animals do what they need, not what we predict). We mentioned this above along with the reference (Landete-Castillejos et al. 2019).

**Reviewer 2 comment:**

| 366–413 | FA context | Tighten; ensure WHO/FAO claim is referenced correctly; remove double period at [36]. | Fix punctuation; ensure every claim has a citation |

**Authors:** Done. We have also greatly reduced the section keeping the essential information (from lines 551-598 to 551-581).

**Reviewer 2 comment:**

Add a short “Limitations” paragraph: single estate; supplementation 300–500 g day⁻¹ is a confounder; age structure differs by month; no diet composition quantified; no serum/bone markers.

**Authors:** We have added this, following reviewer comments “*It is important to bear in mind the constraints of this study, some of which become specific strengths: 1) it is a single estate (but directly comparable in plant composition, climate, and management with previous study 12); 2) the supplementation is common practice in game estates, but departs diet from one based on wild plants alone, and also it is impossible to control exact diet composition; 3) as animals are not picked up in cohorts in a farm, random shooting at distance led to some age structure effects by month*”

# 3. **Methods (lines 415–end).**

**Reviewer 2 comment:**

Instead of Study Plan, you should use Study Area. You need to clearly describe the study area so that readers can easily understand it. Include a map showing the location of your study area. Describe the area in detail, situating the park in relation to the nearby city and the country (Spain), and provide relevant climatic information.

After that, you can add a section titled Data Collection, where you will explain how the data were gathered.

| Lines | Original | Issue | Correction |

**Authors:** We have changed the title of the section to “Study area and design”. We added the following climate description “*The game estate has a continental Mediterranean climate characterized by hot, dry summers and cold winters. Mean annual precipitation is low (~400–450 mm), with rainfall concentrated in autumn and spring and a pronounced summer drought.*”. In addition to this, the paper already had the average temperatures and rainfall for the months included in the study. We think there is no need to include a map just to show that it is in south-central Spain, particularly because the paper is already very long and we have made an effort to shorten.

**Reviewer 2 comment:**

418–420: “38º53’N, 4º17’E” | West longitudes in Spain; likely W | “38°53′N, 4°17′W” |

**Authors:** Corrected, extremely good sight! The first coordinates are 50 km south of Menorca, in the middle of the sea! Sorry for the error.

**Reviewer 2 comment:**

421–426: Plant taxa: “Pistacea Lentiscus…” | Genus/species capitalization and italics | Pistacia lentiscus, Pistacia terebinthus, Cistus ladanifer, Phillyrea angustifolia, Salvia rosmarinus (formerly Rosmarinus officinalis), Quercus ilex, Erica spp., Genista spp. |

**Authors:** Sorry about this. I am zoologist and I always insist my students of proper taxonomical names. I have checked this paper many times, but I overlooked this error of my colleagues. I was not aware of the change in name of rosmary, which looks a lot different from the salvias I know.

**Reviewer 2 comment:**

425–426: “Supplementary food… 300–500 g per deer and day.” | Major confounder; must state composition and brand | Add feed composition (protein, fat, mineral profile), manufacturer, and confirm access was equal across months |

**Authors:** We think this is important, so that despite the length of the paper we have included a new for composition (new table 1).

**Reviewer 2 comment:**

431–433: | Weather means | Fine; note data source | Add data source (station ID) |

**Authors:** Corrected. There is one in Abenójar. We added in lines 119-120: “*(reference weather station Abenójar)*”

**Reviewer 2 comment:**

436–437 : “stalking (sudden-low stress death that did not involve a chase by dogs)” | Hyphenation; neutrally describe | “stalking (selective culling without chase)” |

**Authors:** We have changed the wrong hyphenation to “sudden low-stress death”. As indicated above, low-stress is precise terminology in meat science, animal ethology and other fields. Animals suffer stress, not the least being chased to kill them. This has a number of effects in physiology, and several of them pass onto the meat.

**Reviewer 2 comment:**

452–460: Age determination; decimals with commas | Use decimal points; report agreement | Replace 2,9 with 2.9, etc. Add inter-rater agreement (e.g., 84% exact; Cohen’s κ if available) |

**Authors:** English decimal points placed instead of continental commas. We think the method is sufficiently described (even probably over-explained).

**Reviewer 2 comment:**

462–479: Sampling, pH meter brand | Good; include probe model, calibration details already there | Ensure consistent manufacturer locations; keep T and pH calibration details |

**Authors:** We have added “Instruments” for Crison. We also learned that it has a factory in Spain, so we changed it to “*At the beginning, the pH meter was calibrated using solutions with pH values of 4 and 7 (Crison Instruments, Lainate, Italy and Barcelona, Spain)*”

**Reviewer 2 comment:**

Colour methods :“Objective measures of meat colour [50]…” | Specify device/illuminant/observer/bloom | Add: spectrophotometer model, D65, 10° observer, aperture, 1 h bloom, 3 readings averaged |

**Authors:** We are not sure what the referee means. The model is already in the text, and the rest of the details too (maybe too many): “*(Konica Minolta CM-600d, Osaka, Japan) with a pulsed xenon arc lamp filtered to illuminate D65 lighting conditions…*”

**Reviewer 2 comment:**

| Statistics | Not present in provided lines | PLOS requires full plan | Add: Shapiro–Wilk for normality; Levene for homoscedasticity; one-way ANOVA (factor: month) with Tukey HSD; α = 0.05; report η² and 95% CIs; software/version (R 4.x with packages or SPSS v26) |

**Authors:** The referee is right. We only mentioned normality, but ticked test for homocedasticity in SPSS. We have added “*Normality was assessed using the Shapiro–Wilk test, and homogeneity of variances using Levene’s test. Data were normally distributed with homocedasticity.*”. We had already added Tukey test for another comment above. Lastly, we did not perform an ANOVA, but a general linear model as indicated in the text, which is more general than an ANOVA.

**EDITORIAL COMMENTS:**

When submitting your revision, we need you to address these additional requirements.

1. Please ensure that your manuscript meets PLOS ONE's style requirements, including those for file naming. The PLOS ONE style templates can be found at [https://journals.plos.org/plosone/s/file?id=wjVg/PLOSOne_formatting_sample_main_body.pdf](https://urldefense.com/v3/__https:/journals.plos.org/plosone/s/file?id=wjVg*PLOSOne_formatting_sample_main_body.pdf__;Lw!!D9dNQwwGXtA!UMx4CU6gog_Q9c5hQ3DwtIOmXDlUKZBWcqpeTe1eVYQ5YHVYsvU53UqtkdV3MMuSa5lAqWzE1hTqdA7Iff3UUvQ$) and [https://journals.plos.org/plosone/s/file?id=ba62/PLOSOne_formatting_sample_title_authors_affiliations.pdf](https://urldefense.com/v3/__https:/journals.plos.org/plosone/s/file?id=ba62*PLOSOne_formatting_sample_title_authors_affiliations.pdf__;Lw!!D9dNQwwGXtA!UMx4CU6gog_Q9c5hQ3DwtIOmXDlUKZBWcqpeTe1eVYQ5YHVYsvU53UqtkdV3MMuSa5lAqWzE1hTqdA7IXYx83-k$)

**Authors: Thank you very much.** We will do. Sorry for the delay in replying, but the main researcher addressing the changes, Dr. MPS, had a serious health problem (she is still not back to work), I had several papers written by myself, plus the need to write 2 research projects, and a long etcetera. Another of our coauthors worked partially on these replies, but at the end I had to do a great part of the answering, despite that on my position my duties and workload is exhausting. I am really sorry for the delay and very thankful for the kindness of waiting. I also have to say that in my 32-year scientific career with more than 100 papers in 15 scientific areas, and in my experience as an editor of a Q1 journal, I have never seen such a long report of two reviewers. We thank them for an exhausting effort revising it, which greatly improved the paper, but it also required an enormous amount of time on our side to answer.

**EDITORIAL COMMENT 2**. We note that the grant information you provided in the ‘Funding Information’ and ‘Financial Disclosure’ sections do not match.
When you resubmit, please ensure that you provide the correct grant numbers for the awards you received for your study in the ‘Funding Information’ section.

**Authors:** We have answered this in the second and similar point to be addressed by us.

**EDITORIAL COMMENT 3**. Thank you for stating the following in the Acknowledgments Section of your manuscript:
This research was funded by Spanish research project SBPLY/23/180225/000067 (JCCM regional funds co-financed by European program FEDER) and research project 2022-GRIN-34319 from UCLM University. LC was funded by a postdoctoral contract for scientific excellence of the Plan Propio of UCLM university with Social European Funds (FSE+). T.C., A.G., L.C. and N.A. are members of Instituto de Investigación Sanitaria de Castilla-La Mancha (IDISCAM), Spain. N.A. was funded by the Asociación Española Contra el Cáncer (AECC) to carry out his PhD “Powerful and without side effects: study of the anticancer effect of deer growing antler extract in cell culture and in vivo models (2022-AYUDA-33655)”. The authors thank AECC for their funding support and scientific dissemination of biomedical applications of antler, as well as JCCM and UCLM.

We note that you have provided funding information that is not currently declared in your Funding Statement. However, funding information should not appear in the Acknowledgments section or other areas of your manuscript. We will only publish funding information present in the Funding Statement section of the online submission form.
Please remove any funding-related text from the manuscript and let us know how you would like to update your Funding Statement. Currently, your Funding Statement reads as follows:
This research was funded by Spanish research project SBPLY/23/180225/000067 (JCCM regional funds co-financed by European program FEDER) and research project 2022-GRIN-34319 from UCLM University. LC was funded by a postdoctoral contract for scientific excellence of the Plan Propio of UCLM university with Social European Funds (FSE+). T.C., A.G., L.C. and N.A. are members of Instituto de Investigación Sanitaria de Castilla-La Mancha (IDISCAM), Spain. N.A. was funded by the Asociación Española Contra el Cáncer (AECC) to carry out his PhD “Powerful and without side effects: study of the anticancer effect of deer growing antler extract in cell culture and in vivo models (2022-AYUDA-33655)”. The authors thank AECC for their funding support and scientific dissemination of biomedical applications of antler, as well as JCCM and UCLM.

Please include your amended statements within your cover letter; we will change the online submission form on your behalf.

**Authors:** Thank you very much. We have deleted old version of Acknowledgements, and the new one is this:

*“We thank our coauthor YF for allowing us access to her game estate and cost incurred in donating meat samples for it, all this in addition to her work contribution as a coauthor. We also want to thank regional government in Castilla-La Mancha (JCCM), UCLM university and AECC for their funding (stated in funding section).”*

Project 2022-GRIN-34319 was finished last year, and its acknowledgement will not be accepted. For us, it is mor important to include the new UCLM funding as 2025-GRIN-38458, which we will update in the funding section when resubmitting this article.

**EDITORIAL COMMENT 4.** Please amend your authorship list in your manuscript file to include author Andrés Jose García.

 **Authors:** Thank you very much. I could not understand the error, because he was in the line of authors. The problem is that José is its second name, and because in Spain we have 2 surnames, often we do not state the second name (it is not so important as in the rest of the world because we already have a second surname to distinguish similar first surname authors). Thus, we have forgotten to put his second name in the old version of the article (I did this error often in our 30 year-joint publishing record). We have changed now his name in the article from Andrés García to Andrés José García.

**EDITORIAL COMMENT 5**. Please amend the manuscript submission data (via Edit Submission) to include author Andrés García.

**Authors:** There is only one person: Andrés José García. So we will make sure it is coherent everywhere.

6. If the reviewer comments include a recommendation to cite specific previously published works, please review and evaluate these publications to determine whether they are relevant and should be cited. There is no requirement to cite these works unless the editor has indicated otherwise.

**Authors:** comment not applicable.

Additional Editor Comments: 

Dear Sir, considering the revisions submitted by the reviewers - Major Revision - please comply with the request so that we can proceed.
Sincerely,
JCS
