# Supplementary material for: Effects of season and antler growth on carcass traits and meat quality in wild male red deer harvested by selective stalking
Source: PLoS One. 2026 Jul 8;21(7):e0352456. doi: 10.1371/journal.pone.0352456 (PMC13345254; doi:10.1371/journal.pone.0352456)
Supplement: Attachment — Submitted filename: Manuscript_PONE-D-25-30036R1.docx [file pone.0352456.s004.docx]

**Reviewer 2 – Second Round Report**

**Manuscript:** PONE-D-25-30036R1

**General comment**

The authors have made a serious revision effort and addressed most of my original concerns. The science is sound and the manuscript is close to being publishable. However, I noticed a discrepancy between several corrections described in the response letter and what actually appears in the manuscript body. I list below only the items that still require attention. Once these are fixed, I believe the paper will be ready for publication.

**Mandatory corrections**

1. **Abstract (lines 24–39).** The manuscript body still contains the old abstract ("examines for first time," "culled stress-free," "seems to be caused by," "p = 0.016"). The revised 255-word abstract shown in Editorial Manager must replace this version in the manuscript file.
2. **Section order.** Results currently appear before Materials and Methods (line 89 vs. line 106). PLOS ONE requires: Introduction → Materials and Methods → Results → Discussion → Conclusion. Please reorder accordingly.
3. **Missing Conclusion section.** The response letter states "corrected," but no Conclusion section exists in the manuscript. The text goes from Discussion (ending ~line 595) directly to Author Contributions. Please add a short "5. Conclusion" section.
4. **Line 102: "Hunting period did not affect colour (p > 0.05)."** Hue angle is significant (p = 0.026, Table 1). The revised sentence from the response letter ("hunting period did not affect lightness, redness, yellowness, or chroma, although it did affect Hue angle…") must be inserted here.
5. **Line 117: "Month influenced on the LTL contents of all minerals analysed."** Cu is not significant (p = 0.069). Change "all minerals" to "most minerals" (or "all minerals except Cu").
6. **Table 1, Shear force column header.** Still reads "kg/cm²." The response letter states the unit was changed to N and the ANOVA was re-run. Please ensure the table header, values, and in-text references (e.g., line 111) are consistent either all in Newtons or clearly converted.
7. **Line 114: "Pistacea lentiscus, Pistacea terebinthus."** The correct genus is *Pistacia* (not Pistacea). Also, all species names should be italicised, and "Erica spp" and "Genista spp" need a period: "spp."
8. **Lines 604–606: "Sequence data that support the findings of this study…"** This is a template sentence inappropriate for a meat quality study. Please replace with a correct data availability statement matching the UPMdrive link provided.

**Recommended improvements (optional but encouraged)**

9. **Limitations paragraph (lines 134–140).** Currently placed in Materials and Methods. Consider moving it to the end of the Discussion, just before the new Conclusion section, which is the conventional location.
10. **April Ca value (98.5 mg/100 g, Table 2).** This is 12–17× higher than other months. The biological explanation (skeletal mobilization) is plausible, but adding one sentence confirming that bone-fragment contamination during sampling was ruled out would strengthen confidence in this key result.
11. **Age confounding.** The age data added for June (2.8 ± 0.5 y) vs. September (8.4 ± 2.8 y) in the Discussion (~line 458) is valuable. Consider adding one brief sentence acknowledging that age differences could independently influence mineral or fatty acid composition, beyond the seasonal effect.

**Items from my first review that I accept as resolved**

I accept the authors' arguments on: SD vs. SEM (technically justified for experimental results); absence of additional figures (tables are comprehensive); use of "low-stress" terminology (standard in meat science); DHA reported at p = 0.085 without claiming significance; and self-citation of their antler research (appropriate given their leading expertise). No further action needed on these points.

**Decision: Minor revision.** Items 1–8 are straightforward corrections that should not require re-review.
