# Supplementary material for: Effects of season and antler growth on carcass traits and meat quality in wild male red deer harvested by selective stalking
Source: PLoS One. 2026 Jul 8;21(7):e0352456. doi: 10.1371/journal.pone.0352456 (PMC13345254; doi:10.1371/journal.pone.0352456)
Supplement: Attachment — Submitted filename: Response to reviewers PONE-D-25-30036R1.docx [file pone.0352456.s005.docx]

**Manuscript:** PONE-D-25-30036R1

**Authors response to reviewers.**

We would like to thank both reviewers, particularly the second reviewer, for the great effort to improve our paper. They put an amount of work that exceeds what is normal in a review in these days in which everybody is full of work and duties. Please find in dark red our responses to each of the comments of reviewer 2.

**Reviewer 2 – Second Round Report**

**General comment**

The authors have made a serious revision effort and addressed most of my original concerns. The science is sound and the manuscript is close to being publishable. However, I noticed a discrepancy between several corrections described in the response letter and what actually appears in the manuscript body. I list below only the items that still require attention. Once these are fixed, I believe the paper will be ready for publication.

**Authors:** we thank reviewer very much for his/her effort in improving our paper. Below is a response to his requirements.

**Mandatory corrections**

1. **Abstract (lines 24–39).** The manuscript body still contains the old abstract ("examines for first time," "culled stress-free," "seems to be caused by," "p = 0.016"). The revised 255-word abstract shown in Editorial Manager must replace this version in the manuscript file.

**Authors:** there seems to be an misunderstanding here. I have checked the abstract in the web system (the first that appears in the pdf) with the one in our paper and both coincide, sentence by sentence. I looked for the four examples pointed by the reviewer and none of them is in the paper). In the resrt of the pdf that was sent to the reviewer, the final version of the paper submitted is found in page 191 (where it shows abstract 262 words -we should have deleted the word count, sorry-). Older versions appear on page 50/262 and 97/262, but the correct one is on 191/262. Maybe the reviewer thought page 97/262 was the corrected version, but it is the first review, not the last one.

2. **Section order.** Results currently appear before Materials and Methods (line 89 vs. line 106). PLOS ONE requires: Introduction → Materials and Methods → Results → Discussion → Conclusion. Please reorder accordingly.

**Authors:** This seems to confirm my last comment. In the version that we submitted last, line 196 is material and methods, and on line 297 appear results. Therefore, we already corrected that before submission. Probably the reviewer confounded one of the revised versions with the last revision submitted.

3. **Missing Conclusion section.** The response letter states "corrected," but no Conclusion section exists in the manuscript. The text goes from Discussion (ending ~line 595) directly to Author Contributions. Please add a short "5. Conclusion" section.

**Authors:** the reviewer is correct here and we have added the following conclusion section (highlighted in yellow):

*5. Conclusion*

*Mineral composition displayed a clear seasonal pattern that appeared to be more closely associated with skeletal reserve mobilization during antler development than with changes in diet. In particular, Ca and Mg concentrations were elevated in April, whereas Fe and Zn levels declined in April and June, supporting the hypothesis of mineral mobilization linked to antler growth. Taken together, these results indicate that both seasonality and the physiological demands of antler development significantly influence meat quality and composition in wild male red deer, providing further insight into their nutritional ecology and the characteristics of game meat.*

4. **Line 102: "Hunting period did not affect colour (p > 0.05)."** Hue angle is significant (p = 0.026, Table 1). The revised sentence from the response letter ("hunting period did not affect lightness, redness, yellowness, or chroma, although it did affect Hue angle…") must be inserted here.

**Authors:** Same response as the first 2 points above: that sentence was already in the last version we submitted, lines 310-312. In the pdf, these lines are on page 204 (surprisingly, also on page 157 of an earlie version) of the pdf that was in the PONE web before the current submission. It appears that the reviewer checked one of the very early versions, as this line 102 is found on page 102 of the pdf (and is the version with results before methods).

5. **Line 117: "Month influenced on the LTL contents of all minerals analysed."** Cu is not significant (p = 0.069). Change "all minerals" to "most minerals" (or "all minerals except Cu").

**Authors:** This sentence appears on page 102, as the one above, but same one appears on page 157, and 204. Thus, we have corrected and the sentence is now: “*Month influenced on the Longissimus thoracis et lumborum (LTL) contents of most minerals analysed*”.

6. **Table 1, Shear force column header.** Still reads "kg/cm²." The response letter states the unit was changed to N and the ANOVA was re-run. Please ensure the table header, values, and in-text references (e.g., line 111) are consistent either all in Newtons or clearly converted.

**Authors:** Table 1 shows kg/cm^2^ on page 54 and 101 of the pdf, but on line 305 of page 156 of that same pdf, which is the last version we submitted before, it reads “Shear force, N/cm^2^”, and the values are modified accordingly. This agrees with what we indicated in the response letter: we had changed it according to the request of the reviewer.

7. **Line 114: "Pistacea lentiscus, Pistacea terebinthus."** The correct genus is *Pistacia* (not Pistacea). Also, all species names should be italicised, and "Erica spp" and "Genista spp" need a period: "spp."

**Authors:** change as suggested.

8. **Lines 604–606: "Sequence data that support the findings of this study…"** This is a template sentence inappropriate for a meat quality study. Please replace with a correct data availability statement matching the UPMdrive link provided.

**Authors:** We have changed the sentence to “*Data have been deposited in the UPMdrive*”

**Recommended improvements (optional but encouraged)**

9. **Limitations paragraph (lines 134–140).** Currently placed in Materials and Methods. Consider moving it to the end of the Discussion, just before the new Conclusion section, which is the conventional location.

**Authors:** Changed as suggested. Now on lines 590-596:

*“It is important to bear in mind the constraints of this study, some of which become specific strengths: 1) it is a single estate (but directly comparable in plant composition, climate, and management with previous study [12]); 2) the supplementation is common practice in game estates, but departs diet from one based on wild plants alone, and it is also impossible to control exact diet composition for an animal in the wild; 3) as animals are not picked up in cohorts in a farm, random shooting at distance led to some age structure effects by month.”*

10. **April Ca value (98.5 mg/100 g, Table 2).** This is 12–17× higher than other months. The biological explanation (skeletal mobilization) is plausible, but adding one sentence confirming that bone-fragment contamination during sampling was ruled out would strengthen confidence in this key result.

**Authors:** we have added the following sentence on line 536: “*In this sense, one-fragment contamination during sampling was ruled out.*”

11. **Age confounding.** The age data added for June (2.8 ± 0.5 y) vs. September (8.4 ± 2.8 y) in the Discussion (~line 458) is valuable. Consider adding one brief sentence acknowledging that age differences could independently influence mineral or fatty acid composition, beyond the seasonal effect.

**Authors:** we disagree with this (we cannot agree with everything the referee suggests, despite accepting most things and thanking him for the great effort).
