# Supplementary material for: Effects of season and antler growth on carcass traits and meat quality in wild male red deer harvested by selective stalking
Source: PLoS One. 2026 Jul 8;21(7):e0352456. doi: 10.1371/journal.pone.0352456 (PMC13345254; doi:10.1371/journal.pone.0352456)
Supplement: Peer review history

# Peer review: Decision Letter 0

**DOI:** 10.1371/journal.pone.0352456.r001

**Authors:** Julio Cesar de Souza

11 Nov 2025

PONE-D-25-30036

Effect of hunting month and physiological effort of antler growth on carcass and meat quality of wild red deer males killed stress-free

PLOS ONE

Dear Dr. Landete-Castillejos,

Thank you for submitting your manuscript to PLOS ONE. After careful consideration, we feel that it has merit but does not fully meet PLOS ONE’s publication criteria as it currently stands. Therefore, we invite you to submit a revised version of the manuscript that addresses the points raised during the review process.

==============================

Dear Sir, considering the revisions submitted by the reviewers - Major Revision - please comply with the request so that we can proceed.

Sincerely,

JCS

==============================

Please submit your revised manuscript by Dec 26 2025 11:59PM. If you will need more time than this to complete your revisions, please reply to this message or contact the journal office at plosone@plos.org. When you're ready to submit your revision, log on to https://www.editorialmanager.com/pone/ and select the 'Submissions Needing Revision' folder to locate your manuscript file.

Please include the following items when submitting your revised manuscript:

- A rebuttal letter that responds to each point raised by the academic editor and reviewer(s). You should upload this letter as a separate file labeled 'Response to Reviewers'.
- A marked-up copy of your manuscript that highlights changes made to the original version. You should upload this as a separate file labeled 'Revised Manuscript with Track Changes'.
- An unmarked version of your revised paper without tracked changes. You should upload this as a separate file labeled 'Manuscript'.

If you would like to make changes to your financial disclosure, please include your updated statement in your cover letter. Guidelines for resubmitting your figure files are available below the reviewer comments at the end of this letter.

If applicable, we recommend that you deposit your laboratory protocols in protocols.io to enhance the reproducibility of your results. Protocols.io assigns your protocol its own identifier (DOI) so that it can be cited independently in the future. For instructions see: https://journals.plos.org/plosone/s/submission-guidelines#loc-laboratory-protocols. Additionally, PLOS ONE offers an option for publishing peer-reviewed Lab Protocol articles, which describe protocols hosted on protocols.io. Read more information on sharing protocols at https://plos.org/protocols?utm_medium=editorial-email&utm_source=authorletters&utm_campaign=protocols.

We look forward to receiving your revised manuscript.

Kind regards,

Julio Cesar de Souza, Ph.D.

Academic Editor

PLOS ONE

Journal Requirements:

When submitting your revision, we need you to address these additional requirements.

1. Please ensure that your manuscript meets PLOS ONE's style requirements, including those for file naming. The PLOS ONE style templates can be found at https://journals.plos.org/plosone/s/file?id=wjVg/PLOSOne_formatting_sample_main_body.pdf and https://journals.plos.org/plosone/s/file?id=ba62/PLOSOne_formatting_sample_title_authors_affiliations.pdf

2. We note that the grant information you provided in the ‘Funding Information’ and ‘Financial Disclosure’ sections do not match.

When you resubmit, please ensure that you provide the correct grant numbers for the awards you received for your study in the ‘Funding Information’ section.

3. Thank you for stating the following in the Acknowledgments Section of your manuscript:

This research was funded by Spanish research project SBPLY/23/180225/000067 (JCCM regional funds co-financed by European program FEDER) and research project 2022-GRIN-34319 from UCLM University. LC was funded by a postdoctoral contract for scientific excellence of the Plan Propio of UCLM university with Social European Funds (FSE+). T.C., A.G., L.C. and N.A. are members of Instituto de Investigación Sanitaria de Castilla-La Mancha (IDISCAM), Spain. N.A. was funded by the Asociación Española Contra el Cáncer (AECC) to carry out his PhD “Powerful and without side effects: study of the anticancer effect of deer growing antler extract in cell culture and in vivo models (2022-AYUDA-33655)”. The authors thank AECC for their funding support and scientific dissemination of biomedical applications of antler, as well as JCCM and UCLM.

We note that you have provided funding information that is not currently declared in your Funding Statement. However, funding information should not appear in the Acknowledgments section or other areas of your manuscript. We will only publish funding information present in the Funding Statement section of the online submission form.

Please remove any funding-related text from the manuscript and let us know how you would like to update your Funding Statement. Currently, your Funding Statement reads as follows:

This research was funded by Spanish research project SBPLY/23/180225/000067 (JCCM regional funds co-financed by European program FEDER) and research project 2022-GRIN-34319 from UCLM University. LC was funded by a postdoctoral contract for scientific excellence of the Plan Propio of UCLM university with Social European Funds (FSE+). T.C., A.G., L.C. and N.A. are members of Instituto de Investigación Sanitaria de Castilla-La Mancha (IDISCAM), Spain. N.A. was funded by the Asociación Española Contra el Cáncer (AECC) to carry out his PhD “Powerful and without side effects: study of the anticancer effect of deer growing antler extract in cell culture and in vivo models (2022-AYUDA-33655)”. The authors thank AECC for their funding support and scientific dissemination of biomedical applications of antler, as well as JCCM and UCLM.

Please include your amended statements within your cover letter; we will change the online submission form on your behalf.

4. Please amend your authorship list in your manuscript file to include author Andrés Jose García.

5. Please amend the manuscript submission data (via Edit Submission) to include author Andrés García.

6. If the reviewer comments include a recommendation to cite specific previously published works, please review and evaluate these publications to determine whether they are relevant and should be cited. There is no requirement to cite these works unless the editor has indicated otherwise.

Additional Editor Comments:

Dear Sir, considering the revisions submitted by the reviewers - Major Revision - please comply with the request so that we can proceed.

Sincerely,

JCS

[Note: HTML markup is below. Please do not edit.]

Reviewers' comments:

Reviewer's Responses to Questions

**Comments to the Author**

1. Is the manuscript technically sound, and do the data support the conclusions?

The manuscript must describe a technically sound piece of scientific research with data that supports the conclusions. Experiments must have been conducted rigorously, with appropriate controls, replication, and sample sizes. The conclusions must be drawn appropriately based on the data presented.

Reviewer #1: Yes

Reviewer #2: Partly

**********

2. Has the statistical analysis been performed appropriately and rigorously?

Reviewer #1: Yes

Reviewer #2: Yes

**********

3. Have the authors made all data underlying the findings in their manuscript fully available?

The PLOS Data policy requires authors to make all data underlying the findings described in their manuscript fully available without restriction, with rare exception (please refer to the Data Availability Statement in the manuscript PDF file). The data should be provided as part of the manuscript or its supporting information, or deposited to a public repository. For example, in addition to summary statistics, the data points behind means, medians and variance measures should be available. If there are restrictions on publicly sharing data—e.g. participant privacy or use of data from a third party—those must be specified.

Reviewer #1: Yes

Reviewer #2: Yes

**********

4. Is the manuscript presented in an intelligible fashion and written in standard English?

PLOS ONE does not copyedit accepted manuscripts, so the language in submitted articles must be clear, correct, and unambiguous. Any typographical or grammatical errors should be corrected at revision, so please note any specific errors here.

Reviewer #1: Yes

Reviewer #2: Yes

**********

5. Review Comments to the Author

Please use the space provided to explain your answers to the questions above. You may also include additional comments for the author, including concerns about dual publication, research ethics, or publication ethics. (Please upload your review as an attachment if it exceeds 20,000 characters)

Reviewer #1: ________________________________________

Reviewer Report: PONE-D-25-30036

Manuscript Title: [Effect of hunting month and physiological effort of antler growth on carcass and meat quality of wild red deer males killed stress-free]

Journal: [PLOS ONE]

-----------------------------------------------------------------------------------------------------------

Reviewer Report

Summary of the Research and Overall Impression

The manuscript explores the influence of hunting month and the physiological demands of antler growth on carcass and meat quality traits in wild red deer. The topic is original and highly relevant to wildlife physiology, game meat science, and ecological management. The study addresses a novel research question that integrates seasonality with metabolic and mineral dynamics in deer.

Overall, the work is promising and potentially valuable, but several sections require clearer structuring, refinement of interpretation, and improved scientific precision to meet PLOS ONE standards.

________________________________________

Major Comments

(Lines 27–29) The sentence “This study examines for first time the effects of seasonality…” should be corrected for grammar (e.g., “for the first time”) and revised for clarity. The authors should also state their specific hypotheses or objectives explicitly to strengthen the abstract’s focus.

(Lines 25–39) The abstract currently blends background, results, and interpretation. A more structured flow would improve readability:

1–2 sentences for background/context;

1 sentence for the main aim;

2–3 sentences summarizing key findings with direction and significance;

1 concluding sentence emphasizing novelty or implications.

(Lines 31–34) While including p-values is good practice, ensure consistency (use “p < 0.05” uniformly). Mentioning the general type of statistical test (e.g., ANOVA or mixed model) would help readers appreciate the analytical rigor.

Interpretation vs. Data Presentation (Lines 35–38) Avoid causal phrasing such as “caused by.” Since this is observational research, use softer wording like “may be influenced by” or “is likely associated with” to align with descriptive study conventions.

Minor Comments

• Maintain consistent spacing around parentheses and symbols (e.g., p < 0.05).

• Replace “culled stress-free” with “harvested under minimal stress conditions” for more formal scientific tone.

• Clarify “loin muscle quality” by identifying the specific muscle studied (e.g., Longissimus thoracis et lumborum).

• Strengthen the concluding line by emphasizing novelty, e.g.,

“These findings demonstrate that seasonal dynamics and physiological investment in antler growth jointly influence meat composition in wild red deer, offering new insights into nutritional ecology and game meat quality.”

Introduction

(Lines 45–71) The introduction combines several themes—environmental sustainability, deer biology, antler physiology, and hunting practices—without a clear logical progression. Consider restructuring:

• Paragraph 1: Importance of venison as a sustainable protein source.

• Paragraph 2: Seasonal physiological effects on meat quality.

• Paragraph 3: Mineral mobilization and antler growth.

• Paragraph 4: Hunting context and stress effects.

• Final paragraph: Explicitly state study aims and hypotheses.

(Lines 82–88) The novelty and objectives should be emphasized more clearly to justify the study’s rationale.

(Lines 61–67)The discussion of “cyclic osteoporosis” is interesting but overly detailed for the Introduction. Condense this section and refer readers to the cited review ([11]) for further context.

(Lines 68–71) The statement that antler-derived biomolecules may have anti-aging or anti-cancer properties is speculative. It should be softened (e.g., “It has been suggested that…”) or omitted to maintain scientific objectivity.

Minor Notes

• Define abbreviations such as FA (fatty acids) upon first mention.

• Use the species name “red deer (Cervus elaphus)” consistently.

• Check reference formatting (use either “(1,2)” or “[1,2]” per journal style).

• Ensure that all numerical or quantitative claims are properly cited.

Results

(Lines 132–180) The observed seasonal variations are clearly presented, but biological interpretation should be strengthened. The authors should explicitly link observed trends (e.g., higher carcass yield in September) to nutritional status and antler growth demands, rather than presenting them descriptively.

(Lines 181–210) Mineral variation results (Ca, Mg, Fe, Zn) are intriguing but require deeper explanation. Discuss how these results relate to bone mobilization and antlerogenesis, citing relevant literature on mineral metabolism in cervids.

(Lines 211–250) The section on fatty acid profiles can be condensed. Focus on the biological implications of PUFA/SFA ratios and related health indices, rather than excessive numerical details.

• Ensure uniform significance notation (p < 0.05).

• Clarify if post-hoc corrections were applied for multiple testing.

Minor Suggestions Group related fatty acid indices or mineral results into summary tables to improve clarity and readability.

Discussion

(Lines 189–410) The Discussion is lengthy and partially repetitive. Consider condensing it by removing background information already presented in the Introduction.

Each results subsection (carcass, meat traits, minerals, FA profile) is discussed in isolation; an integrative paragraph linking these findings through nutrition–antler growth–metabolism interactions would enhance coherence.Some statements (e.g., hunter bias, lines 244–250) are speculative and should be rephrased cautiously unless supported by data.

Also, the Discussion relies heavily on the group’s own previous studies [10, 12]. A broader comparison with international studies would improve generalizability.

Minor Adjustments

• Simplify overly long sentences (e.g., lines 300–310).

• Standardize terminology (IMF, intramuscular fat, slaughter BW).

• End with a concise, practical statement highlighting management implications, e.g., “Identifying the optimal hunting period could enhance both meat quality and resource sustainability.”

• Materials and Methods

(Lines 427–460) Although the design is balanced (n = 8 per month), age varies notably among months (~8 years in September vs. ~3 years in others). This age difference is a major confounder that could influence carcass and meat traits. Clarify whether age was statistically controlled (e.g., as a covariate), and if not, acknowledge this limitation explicitly.

(Lines 572–579) The General Linear Model includes only “hunting month” as a fixed factor. Given the complexity of biological variation, age and possibly environmental factors (temperature, rainfall) should be considered covariates. Indicate whether post-hoc tests were adjusted for multiple comparisons (e.g., Tukey HSD).

(Lines 463–466)Specify the time interval between death and muscle sampling, as this directly affects pH and biochemical parameters. Clarify whether carcasses were cooled before sampling or analyzed fresh.

Minor Comments

• Use decimal points consistently (e.g., 2.9 ± 0.7 years).

• Correct typographical error: “cocked meat weight” → “cooked meat weight.”

• Ensure full citation details for AOAC and ISO references (e.g., publication year, method number).

Overall Evaluation

The manuscript presents a valuable contribution to the understanding of seasonal physiology and meat quality in wild red deer. However, revisions are needed to improve structure, refine interpretation, and strengthen statistical transparency. Addressing the confounding effect of age, clarifying methodological details, and condensing the Discussion will substantially enhance the study’s clarity and scientific impact.

Reviewer #2: I. GENERAL COMMENT

The work is solid, with original data and a generally good structure; however, the manuscript lacks a Conclusion section, and the order of sections does not follow standard conventions. Please reorganize it as follows: Introduction, Methods, Results, Discussion, and Conclusion. In addition, make sure to clearly name and number each section and subsection (for example: 1. Introduction; 2. Materials and Methods; 2.1. Study Area; 2.2. Data Collection, etc.). For detailed formatting requirements, please refer to the PLOS ONE submission guidelines.

https://journals.plos.org/plosone/s/submission-guidelines?

1. Lines 1–3

Overlong; “killed stress-free” is emotive; I suggestion you to change the title to this

Effects of season and antler growth on carcass traits and meat quality in wild male red deer harvested by selective stalking

2. Line 4-23: Put it in English

3. Abstract (lines 24–39)

According to the PLOS submission guidelines, the abstract must not exceed 300 words.

https://journals.plos.org/plosone/s/submission-guidelines?

a) Line 25–27: “This study examines for first time…” put this one “This study examines, for the first time, the effects…”

b) Line 28–29: “…on loin muscle quality from 32 adult wild male deer culled stress-free.”

c) Line 30–33: “summer; p < 0.001) … (p = 0.016).” Ensure uniform style: “(p < 0.001) … (p = 0.016).”

d) Line 34: “pH at 72 h post-mortem (pH72)”

e) Line 35–38: “composition seems to be caused by the mineral mobilization…” “mineral composition patterns were consistent with seasonal skeletal mobilization during antler growth: Ca and Mg were higher in April; Fe and Zn were lower in April and June.”

f) Line 38–39: “Thus, we can conclude that both, the season and the effort to grow antlers, affected…” “Both season and antler-growth effort affected meat quality and composition in wild male red deer.”

4. Keywords (line 41–42)

“Fatty acids; meat composition; quality traits; red deer; seasonality.”

5. Introduction (lines 44–88)

a) line 48–54: “In fact, currently, red deer is the main wild game meat…” “deer is” → “deer are”; redundant “in fact, currently”; “carbon fingerprint” “Red deer are a major game meat worldwide, and consumption has increased over the last decade [1,2]. Venison from wild or estate culls has a lower environmental impact and carbon footprint than meat from industrial livestock systems [3].”

b) line 55–60: “Season has an impact… hunting period influences growth [5], [6] …” Clustered numeric citations; use ranges when appropriate; tighten | “Season affects cervid meat quality, particularly in seasonal breeders [4–10]. Hunting period also influences growth and carcass traits [5–7].”

c) line 61–66: “In fact, antlers are the fastest growing tissue and constituting…” Grammar; “skeleton mass” “Antlers are the fastest-growing mammalian appendage and can represent up to 28% of skeletal mass, driving cyclical physiological osteoporosis in spring to mineralize antlers [11]. This may alter meat mineral composition [12].”

d) line 68–71 “the possibility that the anticancer properties…” Off-scope speculation in Intro Delete from Introduction or move as a brief, clearly speculative note to Discussion.

e) line 72–79: “Monteria… thus producing a stressful death), or non-stressful death…” | Emotive language; define neutrally | “Common methods in Spain include Montería (driven hunts with dogs) and stalking (selective culling without chase). Driven hunts can increase pre-mortem stress relative to stalking [12].”

f) line 82–88: Aim paragraph Redundant season labels “We tested the effect of hunting month (September, January, April, June) on carcass and meat traits of wild red deer males culled by stalking, avoiding confounding by hunting stress.”

6. RESULT

Add at least one figure showing seasonal carcass weight/yield with mean ± 95% CI.

Add one figure showing key mineral or FA indices by month.

6.1. Table 1 — BW, carcass, pH, color, composition, cooking, texture

a) Recalculate Chroma (C*) for January; it’s inconsistent with the a* and b* you reported. correct January value (~19). Add footnote: “C* computed from mean a*, b*.”

b) Hue (H): your values are labeled in degrees but look like radians. Either convert to degrees or relabel the unit. Then recheck the p-value.

c) Align your text with the table: don’t claim “no color effect” if Hue is significant. Specify exactly which color variables are/aren’t affected.

d) Shear force: fix the unit. Report Warner–Bratzler in Newtons (or kgf) consistently, not “kg/cm².” If any values were in a different unit, convert and rerun the stats and post-hoc letters.

e) Provide mean ± SD (not just SEM) and name the post-hoc test (e.g., Tukey HSD). PLOS prefers mean ± SD for interpretability

f) f) Line 99, Table 1 must be mentioned in the interpretation.

6.2. Table 2 Minerals (mg/100 g muscle)

a) Don’t say the month affected all minerals. Cu is not significant; say “most minerals.”

b) Investigate the April Ca spike. Verify lab notes, dilutions, and potential bone contamination. If confirmed as an outlier, justify it with QC and a sensitivity analysis, or correct the value if it was a transcription error.

c) Explain the large month-to-month variability in Na (management details like supplemental feed/salt access or transport conditions). Add a brief limitations note if you cannot document it.

d) Report mean ± SD, LOD/LOQ, and any CRM or recovery data used for ICP-OES.

e) Line 115-116: “Potassium (K) was the most abundant micromineral (42.8% of total), followed by P (35.6%) and Na (13.7%).”

6.3. Table 3: Fatty acids in mg/100 g muscle

a) Do not imply a significant difference for DHA on this basis if the p-value is not significant.

b) Standardize isomer labels (e.g., C18:1 t11, not “C 18:1-11t”). Remove stray spaces and unify notation.

c) Acknowledge and explain the mismatch between total FA per 100 g muscle and IMF from Table 1 (different subsamples, FAME→FA conversion, within-muscle variability). Make this explicit in Methods/Discussion.

6.4. Table 4: Total FA (mg/100 g) and aggregate indices

a) Fix number formatting (thousands separator) and consistent “p-value” styling.

b) In the text, remind readers this table is on a muscle basis (mg/100 g), which can yield different significance patterns from percentage basis in Table 5.

6.5. Table 5: Fatty acids as % of total FA (g/100 g FA)

a) Correct the impossible C18:0 percentage in January. A component cannot exceed the class total. Recompute from your mg/100 g data.

b) Verify unusually high C15:1 n-5 values. Check identification against standards, consider coelution, and confirm with additional QC (and revise if misassigned).

c) Clean up nomenclature for trans isomers and spacing.

d) After any correction, recalculate totals, ANOVA, and post-hoc letters as needed.

6.6. Table 6: Indices (percentage basis)

a) These indices depend on Table 5. After you fix Table 5, recompute all indices and confirm significance and letters.

b) Add the explicit formulas for h/H, IA, IT in a footnote with the proper references. Keep notation consistent.

6.7. Table 6 interpretation must do (and currently doesn’t)

1. State the data basis up front.

Say clearly that Table 6 indices are calculated on a percentage basis (g/100 g of total FA, i.e., depend on Table 5), not on mg/100 g muscle. Your text mixes bases elsewhere; don’t do that here.

2. Name the winners/losers for each index, with direction and meaning.

For each line in Table 6, you must explicitly say which month is highest/lowest and what that implies:

o PUFA/SFA: January highest; others much lower. Spell out that this indicates a more favorable lipid profile in January.

o Σn-6: January highest.

o Σn-3: January highest.

o n-6/n-3: January highest; April lowest. Explain the logic: ratio rises in January because n-6 increases more than n-3.

o Long-chain n-3 (EPA+DPA+DHA): January highest; April/June lowest. Note the nutritional relevance.

o Nutritional value (NV = Σ[C12:0+C14:0+C16:0]/Σ[C18:1n-9+C18:2n-6]): April and June highest, January lowest. Add the interpretation: higher NV = less favorable.

o h/H: January maximal. Say explicitly: higher h/H = better.

o IA: January minimal; April/June maximal. Say: lower IA = better.

o IT: January minimal; others higher. Say: lower IT = better.

3. Stop referencing Table 6 for percentages of individual FA.

Your current paragraph drifts into single-FA percentages and even cites Table 6 for that. Table 6 is indices only. Keep individual FA percentages in Table 5 and quantities in Table 3/4.

4. Align claims with p-values.

If you claim an index “differs among months,” you must reference the Table 6 p-value for that index. Do not imply significance where it isn’t shown.

5. Add the formulas and references.

In a footnote (or Methods), include the explicit definitions with citations (Santos-Silva for h/H; Ulbricht & Southgate for IA and IT). If you don’t show formulas, reviewers will assume hand-waving.

6. Acknowledge dependency on Table 5 corrections.

Your indices inherit errors from Table 5. You must say you will recompute the indices and recheck significance and letter codes after fixing Table 5 (e.g., the impossible January C18:0 percentage; the suspiciously high C15:1 n-5 identification).

7. Discussion (lines 189–414)

| Lines | Original | Issue | Correction |

|---|---|---|

| 189–196 | Long consumer intro | Wordy; keep short | Compress to two sentences focusing on supply, seasonality barrier, farmed venison comparison. |

| 213–214 | “we designed current study…” | Article; clarity | “we designed the present study to isolate season effects by culling animals by stalking in all months.” |

| 234–252 | June selection bias argument | Speculative; support with age data already given | Tie to ages (line 459): “September males were older (8.4 ± 2.8 y) than June (2.8 ± 0.5 y), consistent with selection against high-trophy prospects in June; this may partly explain lower BW/carcass yield.” |

| 272–299 | pH interpretation | Fine; trim repetition; emphasize no DFD | Keep core; remove duplicated literature exposition |

| 332–364 | Minerals mechanism | Good idea; but April Ca outlier must be validated | Add limitations: lack of concurrent serum/bone biomarkers; muscle is an indirect proxy |

| 366–413 | FA context | Tighten; ensure WHO/FAO claim is referenced correctly; remove double period at [36].. | Fix punctuation; ensure every claim has a citation |

Add a short “Limitations” paragraph: single estate; supplementation 300–500 g day⁻¹ is a confounder; age structure differs by month; no diet composition quantified; no serum/bone markers.

8. Methods (lines 415–end)

Instead of Study Plan, you should use Study Area. You need to clearly describe the study area so that readers can easily understand it. Include a map showing the location of your study area. Describe the area in detail, situating the park in relation to the nearby city and the country (Spain), and provide relevant climatic information.

After that, you can add a section titled Data Collection, where you will explain how the data were gathered.

| Lines | Original | Issue | Correction |

418–420: “38º53’N, 4º17’E” | West longitudes in Spain; likely W | “38°53′N, 4°17′W” |

421–426: Plant taxa: “Pistacea Lentiscus…” | Genus/species capitalization and italics | Pistacia lentiscus, Pistacia terebinthus, Cistus ladanifer, Phillyrea angustifolia, Salvia rosmarinus (formerly Rosmarinus officinalis), Quercus ilex, Erica spp., Genista spp. |

425–426:“Supplementary food… 300–500 g per deer and day.” | Major confounder; must state composition and brand | Add feed composition (protein, fat, mineral profile), manufacturer, and confirm access was equal across months |

431–433: | Weather means | Fine; note data source | Add data source (station ID) |

436–437 : “stalking (sudden-low stress death that did not involve a chase by dogs)” | Hyphenation; neutrally describe | “stalking (selective culling without chase)” |

452–460 :Age determination; decimals with commas | Use decimal points; report agreement | Replace 2,9 with 2.9, etc. Add inter-rater agreement (e.g., 84% exact; Cohen’s κ if available) |

462–479:Sampling, pH meter brand | Good; include probe model, calibration details already there | Ensure consistent manufacturer locations; keep T and pH calibration details |

Colour methods :“Objective measures of meat colour [50]…” | Specify device/illuminant/observer/bloom | Add: spectrophotometer model, D65, 10° observer, aperture, 1 h bloom, 3 readings averaged |

| Statistics | Not present in provided lines | PLOS requires full plan | Add: Shapiro–Wilk for normality; Levene for homoscedasticity; one-way ANOVA (factor: month) with Tukey HSD; α = 0.05; report η² and 95% CIs; software/version (R 4.x with packages or SPSS v26) |

**********

6. PLOS authors have the option to publish the peer review history of their article (what does this mean?). If published, this will include your full peer review and any attached files.

If you choose “no”, your identity will remain anonymous but your review may still be made public.

**Do you want your identity to be public for this peer review?**  For information about this choice, including consent withdrawal, please see our Privacy Policy.

Reviewer #1: **Yes:** Thamer R. S. Aljubouri

Reviewer #2: No

**********

[NOTE: If reviewer comments were submitted as an attachment file, they will be attached to this email and accessible via the submission site. Please log into your account, locate the manuscript record, and check for the action link "View Attachments". If this link does not appear, there are no attachment files.]

To ensure your figures meet our technical requirements, please review our figure guidelines: https://journals.plos.org/plosone/s/figures

You may also use PLOS’s free figure tool, NAAS, to help you prepare publication quality figures: https://journals.plos.org/plosone/s/figures#loc-tools-for-figure-preparation

NAAS will assess whether your figures meet our technical requirements by comparing each figure against our figure specifications.

Attachment Submitted filename: Reviewer Report PONE-D-25-30036.pdf

Attachment Submitted filename: GENERAL COMMENT.pdf

# Author comment: Author response to Decision Letter 1

**DOI:** 10.1371/journal.pone.0352456.r002

11 Feb 2026

Reviewer Report: PONE-D-25-30036

Manuscript Title: [Effect of hunting month and physiological effort of antler growth

on carcass and meat quality of wild red deer males killed stress-free]

Journal: [PLOS ONE]

Reviewer 1 Report

Summary of the Research and Overall Impression

The manuscript explores the influence of hunting month and the physiological demands of antler growth on carcass and meat quality traits in wild red deer. The topic is original and highly relevant to wildlife physiology, game meat science, and ecological management. The study addresses a novel research question that integrates seasonality with metabolic and mineral dynamics in deer. Overall, the work is promising and potentially valuable, but several sections require clearer structuring, refinement of interpretation, and improved scientific precision to meet PLOS ONE standards.

Major Comments

Authors: We appreciate the reviewer's suggestions and will detail the changes made one by one.

Reviewer 1 comment: (Lines 27–29) The sentence “This study examines for first time the effects of seasonality…” should be corrected for grammar (e.g., “for the first time”) and revised for clarity. The authors should also state their specific hypotheses or objectives explicitly to strengthen the abstract’s focus.

Authors: Corrected. “This study aimed to evaluate, for the first time, how seasonality—assessed by hunting month …”

Reviewer 1 comment: (Lines 25–39) The abstract currently blends background, results, and interpretation. A more structured flow would improve readability:

1–2 sentences for background/context;

1 sentence for the main aim;

2–3 sentences summarizing key findings with direction and significance;

1 concluding sentence emphasizing novelty or implications.

Authors: We have modified the structure and clarified the context of the entire abstract to address the reviewer's suggestion.

Reviewer 1 comment: (Lines 31–34) While including p-values is good practice, ensure consistency (use “p < 0.05” uniformly). Mentioning the general type of statistical test (e.g., ANOVA or mixed model) would help readers appreciate the analytical rigor.

Authors: We have modified the p-values in the abstract and standardised them to p < 0.05.

Reviewer 1 comment: Interpretation vs. Data Presentation (Lines 35–38) Avoid causal phrasing such as “caused by.” Since this is observational research, use softer wording like “may be influenced by” or “is likely associated with” to align with descriptive study conventions.

Authors: We have modified the sentence: “…mineral composition may be more strongly associated with skeletal mobilization during antler growth than with dietary variation: calcium and magnesium concentrations were elevated in April, while iron and zinc levels were reduced in April and June.”

Minor Comments

Reviewer 1 comment: • Maintain consistent spacing around parentheses and symbols (e.g., p < 0.05).

Authors: Corrected

Reviewer 1 comment: • Replace “culled stress-free” with “harvested under minimal stress conditions” for more formal scientific tone.

Authors: Corrected

Reviewer 1 comment: • Clarify “loin muscle quality” by identifying the specific muscle studied (e.g., Longissimus thoracis et lumborum).

Authors: Corrected

Reviewer 1 comment: • Strengthen the concluding line by emphasizing novelty, e.g., “These findings demonstrate that seasonal dynamics and physiological investment in antler growth jointly influence meat composition in wild red deer, offering new insights into nutritional ecology and game meat quality.”

Authors: Included

Introduction

Reviewer 1 comment: (Lines 45–71) The introduction combines several themes—environmental sustainability, deer biology, antler physiology, and hunting practices—without a clear logical progression. Consider restructuring:

• Paragraph 1: Importance of venison as a sustainable protein source.

• Paragraph 2: Seasonal physiological effects on meat quality.

• Paragraph 3: Mineral mobilization and antler growth.

• Paragraph 4: Hunting context and stress effects.

• Final paragraph: Explicitly state study aims and hypotheses.

Authors: Corrected

Reviewer 1 comment: (Lines 82–88) The novelty and objectives should be emphasized more clearly to justify the study’s rationale.

Authors: Corrected.

Reviewer 1 comment: (Lines 61–67) The discussion of “cyclic osteoporosis” is interesting but overly detailed for the Introduction. Condense this section and refer readers to the cited review ([11]) for further context.

Authors: Corrected

Reviewer 1 comment: (Lines 68–71) The statement that antler-derived biomolecules may have anti-aging or anticancer properties is speculative. It should be softened (e.g., “It has been suggested that…”) or omitted to maintain scientific objectivity.

Authors: Corrected

Minor Notes

Reviewer 1 comment: • Define abbreviations such as FA (fatty acids) upon first mention. Authors: Corrected

We have included “Table 4 (new numbering after including table 1 for feed composition). Effect of hunting month on saturated (SFA), monounsaturated (MUFA), and polyunsaturated (PUFA) fatty acid (FA) profile (mg/100 g of muscle) of meat from adult red deer males”

Reviewer 1 comment: • Use the species name “red deer (Cervus elaphus)” consistently.

Authors: Corrected

Reviewer 1 comment: • Check reference formatting (use either “(1,2)” or “[1,2]” per journal style).

Authors: Corrected

Reviewer 1 comment: • Ensure that all numerical or quantitative claims are properly cited.

Authors: Revised

Results

Reviewer 1 comment: (Lines 132–180) The observed seasonal variations are clearly presented, but biological interpretation should be strengthened. The authors should explicitly link observed trends (e.g., higher carcass yield in September) to nutritional status and antler growth demands, rather than presenting them descriptively.

Authors: The section and text lines the referee indicates refers to results. Results have to be descriptive. It is in discussion where we can link results to nutritional status, antler mineral mobilization per season, etc. to interpret the results. PLoS ONE is not a journal that mixes results and discussion in one section.

Reviewer 1 comment: (Lines 181–210) Mineral variation results (Ca, Mg, Fe, Zn) are intriguing but require deeper explanation. Discuss how these results relate to bone mobilization and antlerogenesis, citing relevant literature on mineral metabolism in cervids.

Authors: As in the previous comment, the results section only presents the results, and the interpretation is rather deeply addressed in the discussion (old version: lines 332-364; revised version: lines 524-556).

Reviewer 1 comment: (Lines 211–250) The section on fatty acid profiles can be condensed. Focus on the biological implications of PUFA/SFA ratios and related health indices, rather than excessive numerical details.

Authors: As with mineral content, the biological implications, as well as those regarding meat quality (which is part of the audience of the article) are addressed in the discussion in lines 558-605 of the new version. The results show the actual composition. It may look lengthy for a reader that is not interested in fatty acids, but we also have to think that meat scientists/nutritionist may be interested in a particular fatty acid, and the information has to be complete (particularly considering that now, electronic pdfs do not imply a cost of paper printed, and lengthy data can just be skipped by some readers, unlike in printed journals in the past).

Reviewer 1 comment: • Ensure uniform significance notation (p < 0.05).

Authors: we found that some p were italics and others were not. We have unified all instances now with the same style.

Reviewer 1 comment: Clarify if post-hoc corrections were applied for multiple testing.

Authors: Sorry, the referee is right: we forgot to mention this, but we did a Tukey test. The sentence in lines 278-280 is now “To discriminate which groups differ from others, as shown on tables, a Tukey test was performed.”

Reviewer 1 comment: Minor Suggestions Group related fatty acid indices or mineral results into summary tables to improve clarity and readability.

Discussion

Reviewer 1 comment: (Lines 189–410) The Discussion is lengthy and partially repetitive. Consider condensing it by removing background information already presented in the Introduction.

Authors: We have reduced it in several parts (in the fatty acid section).

Reviewer 1 comment: Each results subsection (carcass, meat traits, minerals, FA profile) is discussed in isolation; an integrative paragraph linking these findings through nutrition–antler growth– metabolism interactions would enhance coherence. Some statements (e.g., hunter bias, lines 244–250) are speculative and should be rephrased cautiously unless supported by data. Also, the Discussion relies heavily on the group’s own previous studies [10, 12]. A broader comparison with international studies would improve generalizability.

Authors: Starting from the end, we compare our results with those international ones in some aspects, but the point is that our own studies were conducted on the same game estate, with the same laboratory equipment and with many confounding variables being equal. Wherever we can, we compare our results with others. An example is the meat characteristics (lines 510-515): “…the shear force observed in the current study was (…) lower than (…) Cawthorn et al. [26] (…) similar to (…) observed by Piaskowska et al. [27] (…) and (…) by Maggiolino et al. [28]”. However, but the effects we are discussed in our article are directly related to previous study in the same game estate with season and type of hunting/stress combined producing a confounding effect. Regarding reference 11 and others by our group, this is the most comprehensive review on antlers, and our group is the leading group in the world in hard antler science (see ranking of TLC as 5th in the world rank here: https://expertscape.com/ex/antlers; the first 4 work in stem cell biology of the growing antler), so that it is not surprising we cite our papers.

Regarding “an integrative paragraph linking these findings through nutrition–antler growth– metabolism interactions would enhance coherence” we use this approach wherever possible, but we cannot fully do this, for example, in fatty acid composition. In addition, there are other approaches that some readers in meat science, for example, may want to see: “how does fatty acid composition relate to that published in meat science studies on NZ farmed deer”, for example.

Regarding statements on hunter bias, we know it is speculative and we actually point it like this, as a possibility, not a proven-fact.

Minor Adjustments

Reviewer 1 comment: • Simplify overly long sentences (e.g., lines 300–310).

Authors: Those sentences are normal. The only ones that are long, but still reasonable are those in 510-515: “As an average, the shear force observed in the current study was 22.2 N, a value much lower than the 30.2 N observed by Cawthorn et al. [26], for the LTL muscle from male wild fallow deer. However, these values were similar to the 22.1 (stags) and 19.2 (hinds) N observed by Piaskowska et al. [27] for the Longissimus lumborum muscle from wild fallow deer and to the 19.2 N observed by Maggiolino et al. [28] as average for the LTL muscle from male wild red deer with different ages at slaughter.” However, if the referee can point exactly a very long sentence or group of sentences, we are ready to shorten them.

Reviewer 1 comment: • Standardize terminology (IMF, intramuscular fat, slaughter BW). Authors: Corrected

Reviewer 1 comment: • End with a concise, practical statement highlighting management implications, e.g., “Identifying the optimal hunting period could enhance both meat quality and resource sustainability.”

• Materials and Methods

Authors: Good suggestion. It is not so easy, as the hunting period is basically autumn-winter, adding a small proportion for population reduction, but we have added this sentence: “Understanding the seasonal effects in meat quality and antler cycle mobilization of minerals may help to take decisions to balance hunting party or population density reduction with meat quality variations for its marketing.”

Reviewer 1 comment: (Lines 427–460) Although the design is balanced (n = 8 per month), age varies notably among months (~8 years in September vs. ~3 years in others). This age difference is a major confounder that could influence carcass and meat traits. Clarify whether age was statistically controlled (e.g., as a covariate), and if not, acknowledge this limitation explicitly.

Authors: The numbers per month are low. We are already short in sample size (but each animal is worth 1200 € or more, so we cannot ask for a larger sample, and adding a covariate requires more observations for the analysis to be acceptable. With these strong constraints, we have tried to explain in the new version adding the following sentence (lines 155-157): “Unfortunately, shooting depends on the opportunity of an animal appearing on view of the hunter, age cannot be easily determined by sight, and thus, it cannot be controlled easily as in a farm”.

Reviewer 1 comment: (Lines 572–579) The General Linear Model includes only “hunting month” as a fixed factor. Given the complexity of biological variation, age and possibly environmental factors (temperature, rainfall) should be considered covariates. Indicate whether post-hoc tests were adjusted for multiple comparisons (e.g., Tukey HSD).

Authors: As explained above, the more variables we add, the more observations we need, therefore we made as appropriate to the sample size as possible. The GLM is a regression not based on multiple comparisons as a set of ANOVAs. However, the reviewer is right in that we used multiple comparisons in tables to show which groups are different from each other, and these are not explained in the methods. We have added this sentence now (lines 278-280): “To discriminate which groups differ from others, as shown on tables, a Tukey test was performed.”

Reviewer 1 comment: (Lines 463–466). Specify the time interval between death and muscle sampling, as this directly affects pH and biochemical parameters. Clarify whether carcasses were cooled before sampling or analyzed fresh.

Authors: We have clarified this now in the following sentence 155-157: “This means that the time elapsed between death and laboratory analysis was less than 24h, and most of the time the samples were stored at about 5ºC”

Minor Comments

Reviewer 1 comment: • Use decimal points consistently (e.g., 2.9 ± 0.7 years).

Authors: Corrected

Reviewer 1 comment: • Correct typographical error: “cocked meat weight” → “cooked meat weight.”

Authors: Corrected

Reviewer 1 comment: • Ensure full citation details for AOAC and ISO references (e.g., publication year, method number).

Authors: Probably the referee means AOCS and ISO. However, in the references are the details he mentions, and these references were already in the MS. If there is something we did not understand, please clarify and we will change it.

56. AOCS. Official Procedure Am 5-04. Rapid determination of oil/fat utilizing high temperature solvent extraction. American Oil Chemist´s Society Urbana, IL, USA. 2005.

53. ISO 937. International standards meat and meat products. Determination of nitrogen content. International Organization for Standardization. Geneva, Switzerland. 1978.

54. ISO 1442. International standards meat and meat products. Determination of moisture content. International Organization for Standardization. Geneva, Switzerland. 1997.

55. ISO 936. International standards meat and meat products. Determination of ash content. International Organization for Standardization. Geneva, Switzerland. 1998.

REVIEWER 2 COMMENTS

I. GENERAL COMMENT

Reviewer 2 comment: The work is solid, with original data and a generally good structure; however, the manuscript lacks a Conclusion section, and the order of sections does not follow standard conventions. Please reorganize it as follows: Introduction, Methods, Results, Discussion, and Conclusion. In addition, make sure to clearly name and number each section and subsection (for example: 1. Introdu

Attachment Submitted filename: Response to reviewers PONE-D-25-30036.docx

# Peer review: Decision Letter 1

**DOI:** 10.1371/journal.pone.0352456.r003

**Authors:** Julio Cesar de Souza

20 Apr 2026

<div>PONE-D-25-30036R1

Effects of season and antler growth on carcass traits and meat quality in wild male red deer harvested by selective stalking

PLOS One

Dear Dr. Landete-Castillejos,

Thank you for submitting your manuscript to PLOS ONE. After careful consideration, we feel that it has merit but does not fully meet PLOS ONE’s publication criteria as it currently stands. Therefore, we invite you to submit a revised version of the manuscript that addresses the points raised during the review process.

Please submit your revised manuscript by Jun 04 2026 11:59PM. If you will need more time than this to complete your revisions, please reply to this message or contact the journal office at plosone@plos.org. When you're ready to submit your revision, log on to https://www.editorialmanager.com/pone/ and select the 'Submissions Needing Revision' folder to locate your manuscript file.

Please include the following items when submitting your revised manuscript:

- A letter that responds to each point raised by the academic editor and reviewer(s). You should upload this letter as a separate file labeled 'Response to Reviewers'.
- A marked-up copy of your manuscript that highlights changes made to the original version. You should upload this as a separate file labeled 'Revised Manuscript with Track Changes'.
- An unmarked version of your revised paper without tracked changes. You should upload this as a separate file labeled 'Manuscript'.

If you would like to make changes to your financial disclosure, please include your updated statement in your cover letter. Guidelines for resubmitting your figure files are available below the reviewer comments at the end of this letter.

If applicable, we recommend that you deposit your laboratory protocols in protocols.io to enhance the reproducibility of your results. Protocols.io assigns your protocol its own identifier (DOI) so that it can be cited independently in the future. For instructions see: https://journals.plos.org/plosone/s/submission-guidelines#loc-laboratory-protocols. Additionally, PLOS ONE offers an option for publishing peer-reviewed Lab Protocol articles, which describe protocols hosted on protocols.io. Read more information on sharing protocols at https://plos.org/protocols?utm_medium=editorial-email&utm_source=authorletters&utm_campaign=protocols.

As the corresponding author, your ORCID iD is verified in the submission system and will appear in the published article. PLOS supports the use of ORCID, and we encourage all coauthors to register for an ORCID iD and use it as well. Please encourage your coauthors to verify their ORCID iD within the submission system before final acceptance, as unverified ORCID iDs will not appear in the published article. *Only*  the individual author can complete the verification step; PLOS staff *cannot*  verify ORCID iDs on behalf of authors.

We look forward to receiving your revised manuscript.

Kind regards,

Julio Cesar de Souza, Ph.D.

Academic Editor

PLOS One

Journal Requirements:

1. If the reviewer comments include a recommendation to cite specific previously published works, please review and evaluate these publications to determine whether they are relevant and should be cited. There is no requirement to cite these works unless the editor has indicated otherwise.

2. Please review your reference list to ensure that it is complete and correct. If you have cited papers that have been retracted, please include the rationale for doing so in the manuscript text, or remove these references and replace them with relevant current references. Any changes to the reference list should be mentioned in the rebuttal letter that accompanies your revised manuscript. If you need to cite a retracted article, indicate the article’s retracted status in the References list and also include a citation and full reference for the retraction notice.

Additional Editor Comments:

Please, I need they attend to the reviewers' suggestions.

Att

[Note: HTML markup is below. Please do not edit.]

Reviewer's Responses to Questions

**Comments to the Author**

1. If the authors have adequately addressed your comments raised in a previous round of review and you feel that this manuscript is now acceptable for publication, you may indicate that here to bypass the “Comments to the Author” section, enter your conflict of interest statement in the “Confidential to Editor” section, and submit your "Accept" recommendation.

Reviewer #1: (No Response)

Reviewer #2: (No Response)

**********

2. Is the manuscript technically sound, and do the data support the conclusions?

The manuscript must describe a technically sound piece of scientific research with data that supports the conclusions. Experiments must have been conducted rigorously, with appropriate controls, replication, and sample sizes. The conclusions must be drawn appropriately based on the data presented.

Reviewer #1: Yes

Reviewer #2: Yes

**********

3. Has the statistical analysis been performed appropriately and rigorously?

Reviewer #1: Yes

Reviewer #2: Yes

**********

4. Have the authors made all data underlying the findings in their manuscript fully available?

The PLOS Data policy requires authors to make all data underlying the findings described in their manuscript fully available without restriction, with rare exception (please refer to the Data Availability Statement in the manuscript PDF file). The data should be provided as part of the manuscript or its supporting information, or deposited to a public repository. For example, in addition to summary statistics, the data points behind means, medians and variance measures should be available. If there are restrictions on publicly sharing data—e.g. participant privacy or use of data from a third party—those must be specified.

Reviewer #1: Yes

Reviewer #2: Yes

**********

5. Is the manuscript presented in an intelligible fashion and written in standard English?

PLOS ONE does not copyedit accepted manuscripts, so the language in submitted articles must be clear, correct, and unambiguous. Any typographical or grammatical errors should be corrected at revision, so please note any specific errors here.

Reviewer #1: Yes

Reviewer #2: Yes

**********

6. Review Comments to the Author

Please use the space provided to explain your answers to the questions above. You may also include additional comments for the author, including concerns about dual publication, research ethics, or publication ethics. (Please upload your review as an attachment if it exceeds 20,000 characters)

Reviewer #1: The manuscript addresses a relevant and interesting topic, providing original data on the interaction between seasonality and antler growth in shaping carcass and meat quality traits in wild red deer. The study is generally well-designed, and the revised version shows clear improvement in structure, formatting, and methodological clarity.

The authors have satisfactorily addressed many of the technical and editorial comments, including corrections to tables, statistical reporting, and organization of the manuscript. The inclusion of a limitations section and clarification of analytical procedures are also appreciated.

However, a few issues still require attention before the manuscript can be considered fully ready for publication. In particular, the role of age as a confounding factor should be more clearly acknowledged and discussed in relation to the results. Additionally, while the Results and Discussion sections are improved, further refinement of the narrative—especially in guiding the reader through key biological implications—would enhance clarity and impact.

In some cases, the interpretation relies on implicit understanding, and more explicit explanation of the main findings (particularly regarding fatty acid indices and mineral patterns) would benefit a broader readership. The manuscript would also gain from slight condensation of repetitive statements and a more balanced integration of relevant international literature.

Overall, the study is scientifically sound and contributes valuable insights to the field. With minor revisions focused on clarity, interpretation, and presentation, the manuscript will be suitable for publication.

Reviewer #2: Reviewer 2 – Second Round Report

Manuscript: PONE-D-25-30036R1

General comment

The authors have made a serious revision effort and addressed most of my original concerns. The science is sound and the manuscript is close to being publishable. However, I noticed a discrepancy between several corrections described in the response letter and what actually appears in the manuscript body. I list below only the items that still require attention. Once these are fixed, I believe the paper will be ready for publication.

Mandatory corrections

1. Abstract (lines 24–39). The manuscript body still contains the old abstract ("examines for first time," "culled stress-free," "seems to be caused by," "p = 0.016"). The revised 255-word abstract shown in Editorial Manager must replace this version in the manuscript file.

2. Section order. Results currently appear before Materials and Methods (line 89 vs. line 106). PLOS ONE requires: Introduction → Materials and Methods → Results → Discussion → Conclusion. Please reorder accordingly.

3. Missing Conclusion section. The response letter states "corrected," but no Conclusion section exists in the manuscript. The text goes from Discussion (ending ~line 595) directly to Author Contributions. Please add a short "5. Conclusion" section.

4. Line 102: "Hunting period did not affect colour (p > 0.05)." Hue angle is significant (p = 0.026, Table 1). The revised sentence from the response letter ("hunting period did not affect lightness, redness, yellowness, or chroma, although it did affect Hue angle…") must be inserted here.

5. Line 117: "Month influenced on the LTL contents of all minerals analysed." Cu is not significant (p = 0.069). Change "all minerals" to "most minerals" (or "all minerals except Cu").

6. Table 1, Shear force column header. Still reads "kg/cm²." The response letter states the unit was changed to N and the ANOVA was re-run. Please ensure the table header, values, and in-text references (e.g., line 111) are consistent either all in Newtons or clearly converted.

7. Line 114: "Pistacea lentiscus, Pistacea terebinthus." The correct genus is Pistacia (not Pistacea). Also, all species names should be italicised, and "Erica spp" and "Genista spp" need a period: "spp."

8. Lines 604–606: "Sequence data that support the findings of this study…" This is a template sentence inappropriate for a meat quality study. Please replace with a correct data availability statement matching the UPMdrive link provided.

Recommended improvements (optional but encouraged)

9. Limitations paragraph (lines 134–140). Currently placed in Materials and Methods. Consider moving it to the end of the Discussion, just before the new Conclusion section, which is the conventional location.

10. April Ca value (98.5 mg/100 g, Table 2). This is 12–17× higher than other months. The biological explanation (skeletal mobilization) is plausible, but adding one sentence confirming that bone-fragment contamination during sampling was ruled out would strengthen confidence in this key result.

11. Age confounding. The age data added for June (2.8 ± 0.5 y) vs. September (8.4 ± 2.8 y) in the Discussion (~line 458) is valuable. Consider adding one brief sentence acknowledging that age differences could independently influence mineral or fatty acid composition, beyond the seasonal effect.

Items from my first review that I accept as resolved

I accept the authors' arguments on: SD vs. SEM (technically justified for experimental results); absence of additional figures (tables are comprehensive); use of "low-stress" terminology (standard in meat science); DHA reported at p = 0.085 without claiming significance; and self-citation of their antler research (appropriate given their leading expertise). No further action needed on these points.

Decision: Minor revision. Items 1–8 are straightforward corrections that should not require re-review.

**********

7. PLOS authors have the option to publish the peer review history of their article (what does this mean?). If published, this will include your full peer review and any attached files.

If you choose “no”, your identity will remain anonymous but your review may still be made public.

**Do you want your identity to be public for this peer review?**  For information about this choice, including consent withdrawal, please see our Privacy Policy.

Reviewer #1: **Yes:** Thamer R. S. Aljubouri

Reviewer #2: **Yes:** LOMBO BALUMA Didier

**********

[NOTE: If reviewer comments were submitted as an attachment file, they will be attached to this email and accessible via the submission site. Please log into your account, locate the manuscript record, and check for the action link "View Attachments". If this link does not appear, there are no attachment files.]

To ensure your figures meet our technical requirements, please review our figure guidelines: https://journals.plos.org/plosone/s/figures

You may also use PLOS’s free figure tool, NAAS, to help you prepare publication quality figures: https://journals.plos.org/plosone/s/figures#loc-tools-for-figure-preparation.

NAAS will assess whether your figures meet our technical requirements by comparing each figure against our figure specifications.

Attachment Submitted filename: Manuscript_PONE-D-25-30036R1.docx

# Author comment: Author response to Decision Letter 2

**DOI:** 10.1371/journal.pone.0352456.r004

29 Apr 2026

Reviewer 2 – Second Round Report

Manuscript: PONE-D-25-30036R1

General comment

The authors have made a serious revision effort and addressed most of my original concerns. The science is sound and the manuscript is close to being publishable. However, I noticed a discrepancy between several corrections described in the response letter and what actually appears in the manuscript body. I list below only the items that still require attention. Once these are fixed, I believe the paper will be ready for publication.

Authors: we thank reviewer very much for his/her effort in improving our paper. Below is a response to his requirements.

Mandatory corrections

1. Abstract (lines 24–39). The manuscript body still contains the old abstract ("examines for first time," "culled stress-free," "seems to be caused by," "p = 0.016"). The revised 255-word abstract shown in Editorial Manager must replace this version in the manuscript file.

Authors: there seems to be an misunderstanding here. I have checked the abstract in the web system (the first that appears in the pdf) with the one in our paper and both coincide, sentence by sentence. I looked for the four examples pointed by the reviewer and none of them is in the paper). In the resrt of the pdf that was sent to the reviewer, the final version of the paper submitted is found in page 191 (where it shows abstract 262 words -we should have deleted the word count, sorry-). Older versions appear on page 50/262 and 97/262, but the correct one is on 191/262. Maybe the reviewer thought page 97/262 was the corrected version, but it is the first review, not the last one.

2. Section order. Results currently appear before Materials and Methods (line 89 vs. line 106). PLOS ONE requires: Introduction → Materials and Methods → Results → Discussion → Conclusion. Please reorder accordingly.

Authors: This seems to confirm my last comment. In the version that we submitted last, line 196 is material and methods, and on line 297 appear results. Therefore, we already corrected that before submission. Probably the reviewer confounded one of the revised versions with the last revision submitted.

3. Missing Conclusion section. The response letter states "corrected," but no Conclusion section exists in the manuscript. The text goes from Discussion (ending ~line 595) directly to Author Contributions. Please add a short "5. Conclusion" section.

Authors: the reviewer is correct here and we have added the following conclusion section (highlighted in yellow):

5. Conclusion

Mineral composition displayed a clear seasonal pattern that appeared to be more closely associated with skeletal reserve mobilization during antler development than with changes in diet. In particular, Ca and Mg concentrations were elevated in April, whereas Fe and Zn levels declined in April and June, supporting the hypothesis of mineral mobilization linked to antler growth. Taken together, these results indicate that both seasonality and the physiological demands of antler development significantly influence meat quality and composition in wild male red deer, providing further insight into their nutritional ecology and the characteristics of game meat.

4. Line 102: "Hunting period did not affect colour (p > 0.05)." Hue angle is significant (p = 0.026, Table 1). The revised sentence from the response letter ("hunting period did not affect lightness, redness, yellowness, or chroma, although it did affect Hue angle…") must be inserted here.

Authors: Same response as the first 2 points above: that sentence was already in the last version we submitted, lines 310-312. In the pdf, these lines are on page 204 (surprisingly, also on page 157 of an earlie version) of the pdf that was in the PONE web before the current submission. It appears that the reviewer checked one of the very early versions, as this line 102 is found on page 102 of the pdf (and is the version with results before methods).

5. Line 117: "Month influenced on the LTL contents of all minerals analysed." Cu is not significant (p = 0.069). Change "all minerals" to "most minerals" (or "all minerals except Cu").

Authors: This sentence appears on page 102, as the one above, but same one appears on page 157, and 204. Thus, we have corrected and the sentence is now: “Month influenced on the Longissimus thoracis et lumborum (LTL) contents of most minerals analysed”.

6. Table 1, Shear force column header. Still reads "kg/cm²." The response letter states the unit was changed to N and the ANOVA was re-run. Please ensure the table header, values, and in-text references (e.g., line 111) are consistent either all in Newtons or clearly converted.

Authors: Table 1 shows kg/cm2 on page 54 and 101 of the pdf, but on line 305 of page 156 of that same pdf, which is the last version we submitted before, it reads “Shear force, N/cm2”, and the values are modified accordingly. This agrees with what we indicated in the response letter: we had changed it according to the request of the reviewer.

7. Line 114: "Pistacea lentiscus, Pistacea terebinthus." The correct genus is Pistacia (not Pistacea). Also, all species names should be italicised, and "Erica spp" and "Genista spp" need a period: "spp."

Authors: change as suggested.

8. Lines 604–606: "Sequence data that support the findings of this study…" This is a template sentence inappropriate for a meat quality study. Please replace with a correct data availability statement matching the UPMdrive link provided.

Authors: We have changed the sentence to “Data have been deposited in the UPMdrive”

Recommended improvements (optional but encouraged)

9. Limitations paragraph (lines 134–140). Currently placed in Materials and Methods. Consider moving it to the end of the Discussion, just before the new Conclusion section, which is the conventional location.

Authors: Changed as suggested. Now on lines 590-596:

“It is important to bear in mind the constraints of this study, some of which become specific strengths: 1) it is a single estate (but directly comparable in plant composition, climate, and management with previous study [12]); 2) the supplementation is common practice in game estates, but departs diet from one based on wild plants alone, and it is also impossible to control exact diet composition for an animal in the wild; 3) as animals are not picked up in cohorts in a farm, random shooting at distance led to some age structure effects by month.”

10. April Ca value (98.5 mg/100 g, Table 2). This is 12–17× higher than other months. The biological explanation (skeletal mobilization) is plausible, but adding one sentence confirming that bone-fragment contamination during sampling was ruled out would strengthen confidence in this key result.

Authors: we have added the following sentence on line 536: “In this sense, one-fragment contamination during sampling was ruled out.”

11. Age confounding. The age data added for June (2.8 ± 0.5 y) vs. September (8.4 ± 2.8 y) in the Discussion (~line 458) is valuable. Consider adding one brief sentence acknowledging that age differences could independently influence mineral or fatty acid composition, beyond the seasonal effect.

Authors: we disagree with this (we cannot agree with everything the referee suggests, despite accepting most things and thanking him for the great effort).

Attachment Submitted filename: Response to reviewers PONE-D-25-30036R1.docx

# Peer review: Decision Letter 2

**DOI:** 10.1371/journal.pone.0352456.r005

**Authors:** Julio Cesar de Souza

10 Jun 2026

Effects of season and antler growth on carcass traits and meat quality in wild male red deer harvested by selective stalking

PONE-D-25-30036R2

Dear Dr. Landete-Castillejos,

We’re pleased to inform you that your manuscript has been judged scientifically suitable for publication and will be formally accepted for publication once it meets all outstanding technical requirements.

Within one week, you’ll receive an e-mail detailing the required amendments. When these have been addressed, you’ll receive a formal acceptance letter and your manuscript will be scheduled for publication.

An invoice will be generated when your article is formally accepted. Please note, if your institution has a publishing partnership with PLOS and your article meets the relevant criteria, all or part of your publication costs will be covered. Please make sure your user information is up-to-date by logging into Editorial Manager at Editorial Manager® and clicking the ‘Update My Information' link at the top of the page. For questions related to billing, please contact billing support.

If your institution or institutions have a press office, please notify them about your upcoming paper to help maximize its impact. If they’ll be preparing press materials, please inform our press team as soon as possible -- no later than 48 hours after receiving the formal acceptance. Your manuscript will remain under strict press embargo until 2 pm Eastern Time on the date of publication. For more information, please contact onepress@plos.org.

Kind regards,

Julio Cesar de Souza, Ph.D.

Academic Editor

PLOS One

Additional Editor Comments (optional):

Ok, Publish.

Reviewers' comments:

Reviewer's Responses to Questions

**Comments to the Author**

1. If the authors have adequately addressed your comments raised in a previous round of review and you feel that this manuscript is now acceptable for publication, you may indicate that here to bypass the “Comments to the Author” section, enter your conflict of interest statement in the “Confidential to Editor” section, and submit your "Accept" recommendation.

Reviewer #3: All comments have been addressed

Reviewer #4: (No Response)

**********

2. Is the manuscript technically sound, and do the data support the conclusions?

The manuscript must describe a technically sound piece of scientific research with data that supports the conclusions. Experiments must have been conducted rigorously, with appropriate controls, replication, and sample sizes. The conclusions must be drawn appropriately based on the data presented.

Reviewer #3: Yes

Reviewer #4: Yes

**********

3. Has the statistical analysis been performed appropriately and rigorously?

Reviewer #3: Yes

Reviewer #4: Yes

**********

4. Have the authors made all data underlying the findings in their manuscript fully available?

The PLOS Data policy requires authors to make all data underlying the findings described in their manuscript fully available without restriction, with rare exception (please refer to the Data Availability Statement in the manuscript PDF file). The data should be provided as part of the manuscript or its supporting information, or deposited to a public repository. For example, in addition to summary statistics, the data points behind means, medians and variance measures should be available. If there are restrictions on publicly sharing data—e.g. participant privacy or use of data from a third party—those must be specified.

Reviewer #3: Yes

Reviewer #4: Yes

**********

5. Is the manuscript presented in an intelligible fashion and written in standard English?

PLOS ONE does not copyedit accepted manuscripts, so the language in submitted articles must be clear, correct, and unambiguous. Any typographical or grammatical errors should be corrected at revision, so please note any specific errors here.

Reviewer #3: Yes

Reviewer #4: Yes

**********

6. Review Comments to the Author

Please use the space provided to explain your answers to the questions above. You may also include additional comments for the author, including concerns about dual publication, research ethics, or publication ethics. (Please upload your review as an attachment if it exceeds 20,000 characters)

Reviewer #3: This study systematically evaluates the effects of hunting months on carcass characteristics, meat quality, mineral content, and fatty acid composition of wild male red deer, with particular emphasis on the potential role of antler growth on mineral metabolism. The study design is innovative, especially in isolating seasonal effects under controlled hunting stress. The data are robust, and the analytical methods are appropriate. The overall scientific quality is high and suitable for publication in PLOS ONE. However, several issues need to be addressed before acceptance.

1. The authors acknowledge that ages differed significantly between months (e.g., September: 8.4 yr; June: 2.8 yr). However, the potential independent effect of age on meat quality, mineral, and fatty acid profiles is not adequately discussed or controlled. The authors explicitly state they “disagree” with the reviewer’s suggestion to add a sentence acknowledging age effects, which weakens the rigor of the conclusions.

Suggestion: At a minimum, add a sentence in the Limitations section, such as: “Although age may independently influence certain meat quality traits, its effect cannot be fully separated from seasonal and antler‑growth effects in this field‑based study.”

2. References [29] and [30] are duplicated and both numbered “29.”

3. In line 536� “one‑fragment contamination” should be “bone‑fragment...”.

4. It is suggest to provide additional verification for the high Ca value. State clearly that this is a true biological difference and support it with literature.

This is a well‑designed, data‑rich, and practically relevant field study. The authors’ investigation of seasonal and antler‑growth effects on venison quality provides valuable insights for game meat management. The current version has been substantially improved, and the remaining issues are minor. Once these are addressed, the manuscript will fully meet the publication standards of PLOS ONE.

Reviewer #4: I would like to congratulate the authors on their excellent work. Overall, this is a well-conducted study with solid scientific merit that contributes valuable information to the field. My comments are mainly editorial in nature and are intended to improve the fluency, readability, and overall flow of the manuscript.

1. Introduction

lines 74-76: The frequent use of parentheses can disrupt the reading flow. I recommend using commas instead, where appropriate, to enhance readability. "The most common forms of hunting deer in Spain are

the traditional type known as Monteria, in which deer are chased by dogs towards the

hunter’s line, thus producing a stressful death; or non-stressful death by selective..."

lines 77-79: Same above. "This last hunting method form is known as stalking and implies a sudden low stress death, which is also common in most countries."

lines 86-88: This should belong in the discussion rather than introduction "This is the first study to assess the influence of the four seasons of the year on meat quality of deer killed by stalking."

lines 97-98: The use of em dashes (—) is often associated with AI-generated text. I suggest replacing them with commas, where appropriate, to improve readability and maintain a more conventional scientific writing style.

Lastly, please remove the word count indicated next to the “Abstract” heading in the manuscript version.

**********

7. PLOS authors have the option to publish the peer review history of their article (what does this mean?). If published, this will include your full peer review and any attached files.

If you choose “no”, your identity will remain anonymous but your review may still be made public.

**Do you want your identity to be public for this peer review?**  For information about this choice, including consent withdrawal, please see our Privacy Policy.

Reviewer #3: No

Reviewer #4: **Yes:** Sofia Silva La Rocca de Freitas

**********

# Editor report: Acceptance letter

**DOI:** 10.1371/journal.pone.0352456.r006

**Authors:** Julio Cesar de Souza

PONE-D-25-30036R2

PLOS One

Dear Dr. Landete-Castillejos,

I'm pleased to inform you that your manuscript has been deemed suitable for publication in PLOS One. Congratulations! Your manuscript is now being handed over to our production team.

At this stage, our production department will prepare your paper for publication. This includes ensuring the following:

* All references, tables, and figures are properly cited

* All relevant supporting information is included in the manuscript submission,

* There are no issues that prevent the paper from being properly typeset

You will receive further instructions from the production team, including instructions on how to review your proof when it is ready. Please keep in mind that we are working through a large volume of accepted articles, so please give us a few days to review your paper and let you know the next and final steps.

Lastly, if your institution or institutions have a press office, please let them know about your upcoming paper now to help maximize its impact. If they'll be preparing press materials, please inform our press team within the next 48 hours. Your manuscript will remain under strict press embargo until 2 pm Eastern Time on the date of publication. For more information, please contact onepress@plos.org.

You will receive an invoice from PLOS for your publication fee after your manuscript has reached the completed accept phase. If you receive an email requesting payment before acceptance or for any other service, this may be a phishing scheme. Learn how to identify phishing emails and protect your accounts at https://explore.plos.org/phishing.

If we can help with anything else, please email us at customercare@plos.org.

Thank you for submitting your work to PLOS ONE and supporting open access.

Kind regards,

PLOS ONE Editorial Office Staff

on behalf of

Dr. Julio Cesar de Souza

Academic Editor

PLOS One
